# Cell-surface marker discovery for lung cancer

**DOI:** 10.18632/oncotarget.23009

**Published:** 2017-12-07

**Authors:** Allison S. Cohen, Farah K. Khalil, Eric A. Welsh, Matthew B. Schabath, Steven A. Enkemann, Andrea Davis, Jun-Min Zhou, David C. Boulware, Jongphil Kim, Eric B. Haura, David L. Morse

**Affiliations:** ^1^ Department of Cancer Imaging and Metabolism, H. Lee Moffitt Cancer Center and Research Institute, Tampa, FL, USA; ^2^ Department of Anatomic Pathology, H. Lee Moffitt Cancer Center and Research Institute, Tampa, FL, USA; ^3^ Biomedical Informatics Shared Resource, H. Lee Moffitt Cancer Center and Research Institute, Tampa, FL, USA; ^4^ Department of Cancer Epidemiology, H. Lee Moffitt Cancer Center and Research Institute, Tampa, FL, USA; ^5^ Molecular Genomics Shared Resource, H. Lee Moffitt Cancer Center and Research Institute, Tampa, FL, USA; ^6^ Biostatistics Shared Resource, H. Lee Moffitt Cancer Center and Research Institute, Tampa, FL, USA; ^7^ Department of Biostatistics, H. Lee Moffitt Cancer Center and Research Institute, Tampa, FL, USA; ^8^ Department of Thoracic Oncology, H. Lee Moffitt Cancer Center and Research Institute, Tampa, FL, USA; ^9^ Department of Oncologic Sciences, College of Medicine, University of South Florida, Tampa, FL, USA; ^10^ Department of Physics, College of Arts and Sciences, University of South Florida, Tampa, FL, USA

**Keywords:** lung cancer, cell-surface, target biomarker, targeted therapeutics, molecular imaging

## Abstract

Lung cancer is the leading cause of cancer deaths in the United States. Novel lung cancer targeted therapeutic and molecular imaging agents are needed to improve outcomes and enable personalized care. Since these agents typically cannot cross the plasma membrane while carrying cytotoxic payload or imaging contrast, discovery of cell-surface targets is a necessary initial step. Herein, we report the discovery and characterization of lung cancer cell-surface markers for use in development of targeted agents. To identify putative cell-surface markers, existing microarray gene expression data from patient specimens were analyzed to select markers with differential expression in lung cancer compared to normal lung. Greater than 200 putative cell-surface markers were identified as being overexpressed in lung cancers. Ten cell-surface markers (CA9, CA12, CXorf61, DSG3, FAT2, GPR87, KISS1R, LYPD3, SLC7A11 and TMPRSS4) were selected based on differential mRNA expression in lung tumors vs. non-neoplastic lung samples and other normal tissues, and other considerations involving known biology and targeting moieties. Protein expression was confirmed by immunohistochemistry (IHC) staining and scoring of patient tumor and normal tissue samples. As further validation, marker expression was determined in lung cancer cell lines using microarray data and Kaplan–Meier survival analyses were performed for each of the markers using patient clinical data. High expression for six of the markers (CA9, CA12, CXorf61, GPR87, LYPD3, and SLC7A11) was significantly associated with worse survival. These markers should be useful for the development of novel targeted imaging probes or therapeutics for use in personalized care of lung cancer patients.

## INTRODUCTION

Lung cancer is the second leading cause of cancer and the leading cause of cancer deaths in both men and women in the United States [1, 2]. Although the mortality rate for lung cancer has declined over the last several decades, the overall 5-year survival rate has not substantially improved over the last 30 years [1, 2]. The majority of lung cancers are diagnosed at a distant stage (57%) [1]. Only 16% of lung cancers are diagnosed at a localized stage, for which the 5-year survival rate is 55% [1, 2]. The five year survival rate decreases for regional and distant cancers (28% and 4%, respectively) [1, 2]. For all stages combined, the five year survival rate is only 18% [1, 2]. Thus, there is a need for new ways to diagnose and treat this disease to improve clinical outcomes.

Early detection of lung cancer improves the patient’s chance of survival. Computed tomography (CT) is the most commonly used modality for lung cancer early detection, staging, treatment evaluation and follow-up [3–5]. Based on the results of the National Lung Screening Trial (NLST), screening by low-dose helical CT has been recommended for the early detection of lung cancer; however this only applies to high risk current and former smokers [2, 3, 6]. Currently, low-dose CT (LDCT) is the only approved method for lung cancer screening [7]. LDCT is useful for detecting small peripheral masses but other techniques are needed for tumors that arise in the central airways [8]. There is also a need for improved methods to discriminate malignant from benign lesions [3, 5]. Positron emission tomography (PET) with ^18^F-fluorodeoxyglucose (^18^F-FDG) can be used for metabolic imaging of lung cancer [3, 5, 9, 10]. It is useful for the detection of metastases and discrimination of malignant from benign lesions [5, 9, 10]. However, other abnormalities including inflammation and infection, can also be observed using ^18^F-FDG PET resulting in false positives [3–5, 9, 10]. Other PET tracers based on alternate pathways, such as proliferation and amino acid uptake, are currently being studied for use in lung cancer [3]. Magnetic resonance imaging (MRI) is used only for limited applications but investigations are being conducted to potentially expand the utility of MRI in the management of lung cancer [3–6]. Autofluorescence is used during bronchoscopy to identify precancerous and cancerous lesions and post-operatively to detect recurrence [3, 11, 12]. However, the current approaches lack specificity due to false positives resulting from other abnormalities such as inflammation [3, 11, 12].

While it is unlikely that molecular imaging agents are practical for use in lung cancer screening, development of novel lung cancer targeted molecular imaging agents has potential to address a number of clinical needs in the diagnosis and management of lung cancer and to augment the personalized care of patients. Since ^18^F-FDG PET imaging is not reliable in the context of inflammation, a lung-cancer specific PET imaging tracer is needed for use in this context, e.g., following surgery or radiation therapy [13]. A lung cancer specific PET tracer could also potentially be used to better distinguish malignant from benign nodules of the lung, which is an unmet clinical need that could improve early detection of lung cancer [14]. Additionally, imaging biomarkers that can non-invasively provide predictive or prognostic information are needed to improve the clinical management of lung cancer [15]. Development of fluorescently labeled lung cancer specific agents could improve early detection via fluorescence bronchoscopy. Such lung cancer targeted fluorescent agents could also be used intraoperatively for margin detection and identification of mediastinal lymph nodes that contain metastases [16].

In recent years, kinase targeted therapies have been developed that have shown improved efficacy in treatment of lung cancer compared to standard chemotherapy, e.g., epidermal growth factor receptor (EGFR) tyrosine kinase inhibitors [17] and anaplastic lymphoma kinase (ALK) inhibitors [18]. Immune checkpoint inhibitors, e.g. anti-PD1 and anti-CTLA-4, are another class of targeted therapies that have shown efficacy in treatment of lung cancer [19]. However, these new targeted treatments are only applicable to a fraction of patients, and development of resistance and recurrence has been a considerable problem in patients that do respond [20, 21]. Studies involving combination therapies have demonstrated increased efficacy and it has been proposed that combinations of therapies that target distinct pathways or mechanisms could increase the period of disease-free survival, or even be curative [22, 23]. However, current targeted therapies are associated with systemic toxicities, lowering the potential for effective combinations. Hence, novel targeted therapies that have low systemic toxicity are needed for use in combination with the existing toolbox of therapies. In addition, companion imaging agents are needed to identify patients that are likely to respond to the corresponding targeted therapy and to non-invasively follow treatment response.

To successfully implement the personalized treatment of lung cancer, molecular imaging agents and targeted therapeutics are needed that can detect the tumor with high specificity and selectivity. Since targeting moieties conjugated to imaging contrast or therapeutic agents cannot cross the plasma membrane, development of agents that target cell-surface markers that are differentially expressed on lung tumors relative to normal tissues or benign lesions is a rational approach toward achieving this objective. Thus, the identification and comparison of cell-surface markers is a crucial first step in the development of novel cancer-specific molecular imaging agents and targeted therapeutics. We have previously identified and validated novel bona fide cancer cell-surface markers by mRNA expression profiling and immunohistochemistry (IHC) of colon, melanoma, pancreatic and breast cancer patient tissue samples [24–30]. We have also developed imaging agents that target these identified tumor cell-surface markers [24, 25, 29, 31–34].

The goal of the current work was to identify a set of cell-surface markers that cover a broad range of lung cancers and analyze the expression of these markers in relation to survival of lung cancer patients. Once determined, such markers may be useful targets for the development of lung cancer targeted imaging and therapeutic agents.

## RESULTS

### Cell-surface marker identification

Our goal was to identify cell-surface markers that can be used for targeted agent development. However, different classes of targeted agent require different metrics for selection. For example, molecular imaging agents typically deliver tracer levels of radioactivity or non-toxic payloads for image contrast. In this case, the most important metric is target expression in tumor relative to surrounding normal lung tissue. Alternately, targeted therapeutic agents can deliver cytotoxic payloads or inhibit pathways that are important for normal cellular functions. Hence, marker discovery for targeted therapy requires evaluation of expression in tumor versus expression in a range of tissues that are of concern for systemic toxicity.

Gene expression profiling was performed using mRNA expression microarray data from patient samples of lung cancer and normal tissues. Available data sets were evaluated for quality, compiled, normalized and a MarkerScore determined for ranking differential expression in tumor relative to normal (see Methods). The probesets were intersected with a list of potential surface accessible gene products to annotate the target location and filter the data set, yielding a set of 11,838 potential surface accessible probesets for further analysis. Gene expression data for these probesets were sorted by MarkerScore using Excel 2010, and the list was analyzed for probesets exhibiting differentially high expression in lung tumors relative to normal lung tissue samples as determined using a combination of statistical tests described in the Methods. This resulted in a list of 360 probesets (282 genes) (Supplementary Table 1). Note that the number of probesets does not correspond to the number of genes, since several genes are detected by multiple probesets in the arrays. Our cell-surface list includes some genes that are membrane associated but do not have cell-surface domains, e.g. code for proteins that are secreted, are associated with the cytoplasmic side of the plasma membrane or with internal membranes only. We reviewed the literature for the list of 282 genes and 268 probesets (208 genes) were identified that likely have cell-surface domains (annotated as 1 in the Cell Membrane column in Supplementary Table 1). These 208 genes were evaluated for potential use as lung cancer specific cell-surface markers based on intensity and breadth of expression among the lung cancer samples relative to their differentially low expression in non-neoplastic lung tissue samples.

In addition to higher expression in tumor samples relative to normal lung samples, expression in other tissues associated with toxicity and clearance, e.g. liver, kidney, heart, etc., was also considered and markers that were expressed in these tissues were de-emphasized. From the ranked list, 10 markers were selected for further evaluation: CA9, CA12, CXorf61, DSG3, FAT2, GPR87, KISS1R, LYPD3, SLC7A11 and TMPRSS4. Five of these markers, CXorf61, DSG3, FAT2, GPR87, and LYPD3, were selected based primarily on their high and broad expression among the lung cancer samples relative to normal lung. Additional markers were selected based on their profile and that there are currently available molecular imaging probes targeting these markers (CA9, CA12, KISS1R and SLC7A11) [25, 35–70]. KISS1R and TMPRSS4 have known high affinity ligands and inhibitors, respectively, for potential use in targeting [71–80]. Despite its relatively low ranking based on marker score, CA9 was selected for further investigation due to an availability of high affinity inhibitors for imaging [49–51] and its general applicability among several cancer types, in addition to lung cancer, including cancers of the brain, breast, cervix, colon, head and neck, kidney, ovaries, and pancreas [25, 35, 81–83].

**Figure 1:**
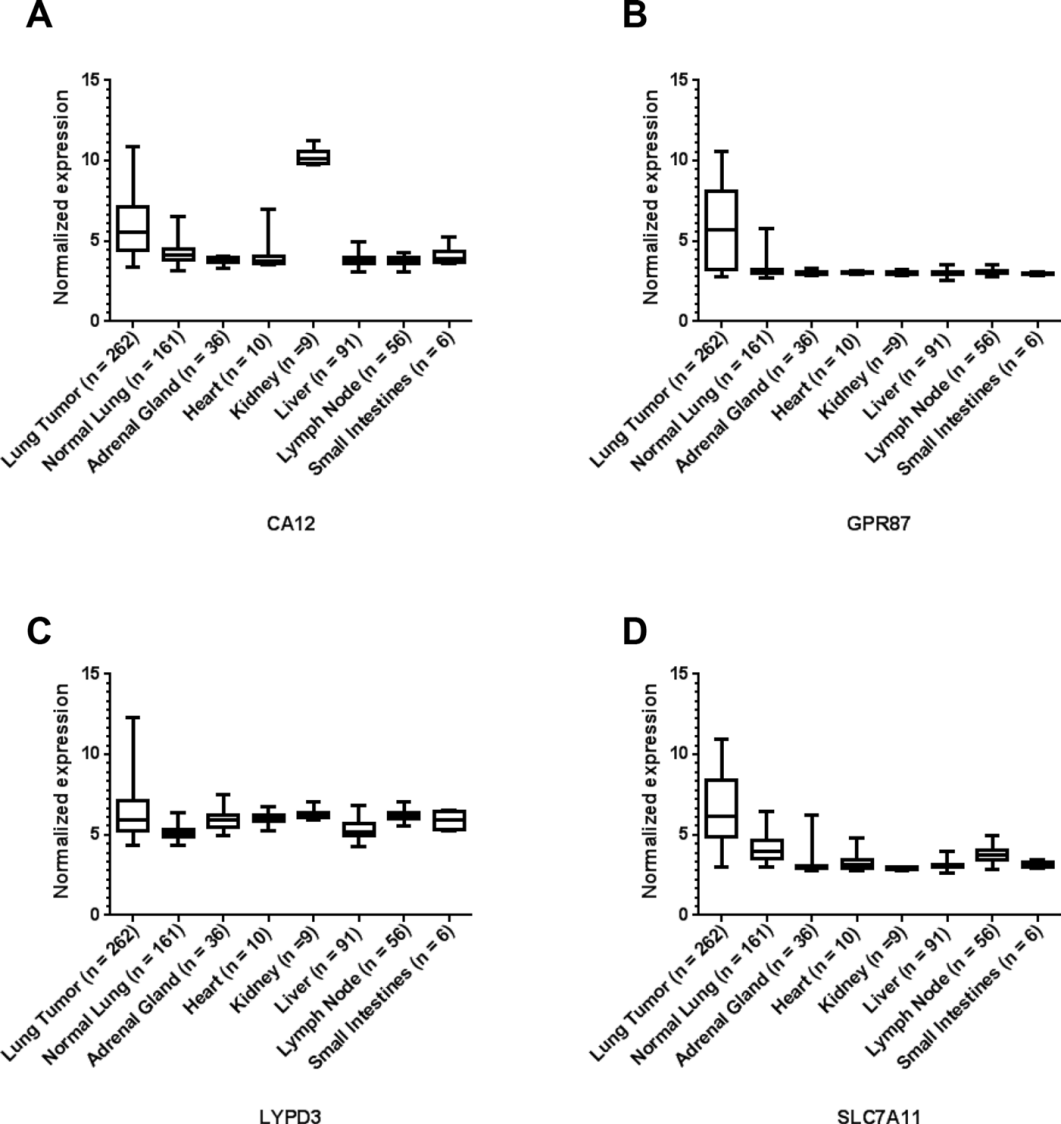
Representative microarray mRNA expression profiles for four of the selected lung cancer cell-surface markers in patient specimens of normal lung, lung tumors and other normal tissues: CA12 (**A**), GPR87 (**B**), LYPD3 (**C**), and SLC7A11 (**D**). Values are presented as whisker/box plots with whiskers representing the full range of values, the bottom and top of the boxes represent the 25th and 75th percentile, and middle lines represent the median.

Figure 1 shows representative mRNA expression profiles of four of the selected markers in patient samples; the mRNA expression profiles for the remaining six selected markers are shown in Supplementary Figure 1. For each of these markers, the mRNA expression is significantly higher in the lung tumor samples in comparison to the normal lung samples (*p* < 0.0001) (Table 1 and Supplementary Tables 2–11). However, none of these markers are expressed at a high level in 100% of the lung tumor samples. Nevertheless, for each marker, there are a percentage of cancer cases with very high expression relative to normal lung. Therefore, a combination of markers may be required to cover all types of lung cancer.

**Table 1:** Adjusted *p* values by Dunnett’s multiple comparisons for lung tumor (control) versus normal tissues.

Tissue type	CA9	CA12	CXorf61	DSG3	FAT2	GPR87	KISS1R	LYPD3	SLC7A11	TMPRSS4
Normal Lung	**<0.0001**	**<0.0001**	**<0.0001**	**<0.0001**	**<0.0001**	**<0.0001**	**<0.0001**	**<0.0001**	**<0.0001**	**<0.0001**
Adrenal Gland	**<0.0001**	**<0.0001**	**<0.0001**	**<0.0001**	**0.017**	**<0.0001**	**<0.0001**	0.15	**<0.0001**	**<0.0001**
Heart	0.88	**<0.0001**	**0.0118**	**0.0161**	**0.0239**	**<0.0001**	**<0.0001**	0.92	**<0.0001**	**<0.0001**
Kidney	0.53	**<0.0001**	**0.0094**	**0.0136**	0.13	**<0.0001**	**<0.0001**	>0.99	**<0.0001**	**<0.0001**
Liver	**0.008**	**<0.0001**	**<0.0001**	**<0.0001**	**<0.0001**	**<0.0001**	**<0.0001**	**<0.0001**	**<0.0001**	**<0.0001**
Lymph Node	**<0.0001**	**<0.0001**	**<0.0001**	**<0.0001**	**<0.0001**	**<0.0001**	**<0.0001**	0.96	**<0.0001**	**<0.0001**
Small Intestines	**0.0039**	**0.0007**	0.13	0.13	0.18	**0.0001**	**<0.0001**	0.85	**<0.0001**	0.14

The mRNA expression of these markers in organs involved in toxicity and clearance was also evaluated. The expression of the markers GPR87, KISS1R and SLC7A11 are significantly higher in the lung tumors than all of the other normal organs examined (Table 1, Supplementary Tables 7, 8 and 10, and Figures 1B and 1D and Supplementary Figure 1E). The expression of the other markers are either significantly higher or show no statistical difference for the tumor in comparison to the other organs in all cases except two (Figure 1 and Supplementary Figure 1, and Table 1 and Supplementary Tables 2–11). In the case of CA12, the expression is significantly higher in the kidney than in the lung tumors (*p* < 0.0001) (Figure 1A and Supplementary Table 3). For CA9, the expression is significantly higher in the small intestines than in the lung tumors (*p* = 0.0039) (Supplementary Figure 1A and Supplementary Table 2).

**Figure 2:**
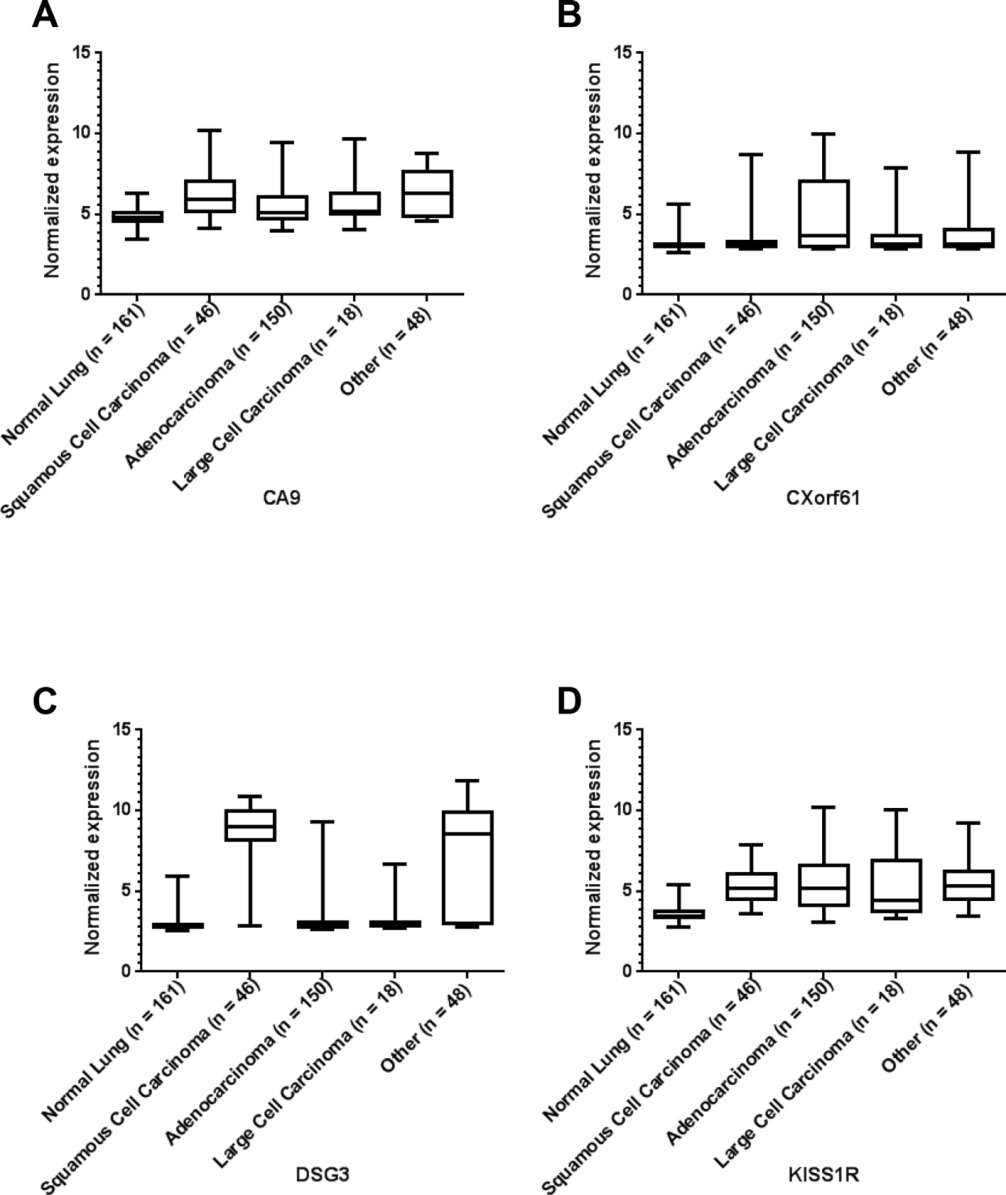
Representative microarray mRNA expression profiles for four of the selected markers in patient specimens of normal lung and lung tumors of various lung cancer histologies: CA9 (**A**), CXorf61 (**B**), DSG3 (**C**), and KISS1R (**D**). Values are presented as whisker/box plots with whiskers representing the full range of values, the bottom and top of the boxes represent the 25^th^ and 75^th^ percentile, and the middle lines represent the median.

In addition to the analysis of marker expression for all types of lung cancer, we also analyzed the expression of each of the markers for the three main histological classes of non-small cell lung cancer (NSCLC) (with a sample number *n* ≥ 3): adenocarcinoma, large cell carcinoma and squamous cell carcinoma (SCC). All remaining mRNA data were combined as “other”. See Figure 2 and Supplementary Figure 2. Statistical differences of the different histological classes relative to normal lung were reported as *p*-values in Table 2 and Supplementary Tables 12–21. Statistical differences among the different histological classes are reported in Supplementary Tables 22–31. Interestingly, some markers are significantly higher in all NSCLC sub-classes relative to normal lung, while other markers have differential expression among the sub-classes.

**Table 2:** Adjusted *p* values by Dunnett’s multiple comparisons for normal lung (control) versus lung cancer histologies

Cancer type	CA9	CA12	CXorf61	DSG3	FAT2	GPR87	KISS1R	LYPD3	SLC7A11	TMPRSS4
Squamous Cell Carcinoma	**<0.0001**	**<0.0001**	0.29	**<0.0001**	**<0.0001**	**<0.0001**	**<0.0001**	**<0.0001**	**<0.0001**	**<0.0001**
Adenocarcinoma	**<0.0001**	**<0.0001**	**<0.0001**	0.08	0.48	**<0.0001**	**<0.0001**	**<0.0001**	**<0.0001**	**<0.0001**
Large Cell Carcinoma	**0.0003**	0.32	0.67	0.95	>0.99	0.95	**<0.0001**	0.55	**<0.0001**	0.57
Other	**<0.0001**	**<0.0001**	**0.0005**	**<0.0001**	**<0.0001**	**<0.0001**	**<0.0001**	**<0.0001**	**<0.0001**	**<0.0001**

### Confirmation of marker protein expression

**Figure 3:**
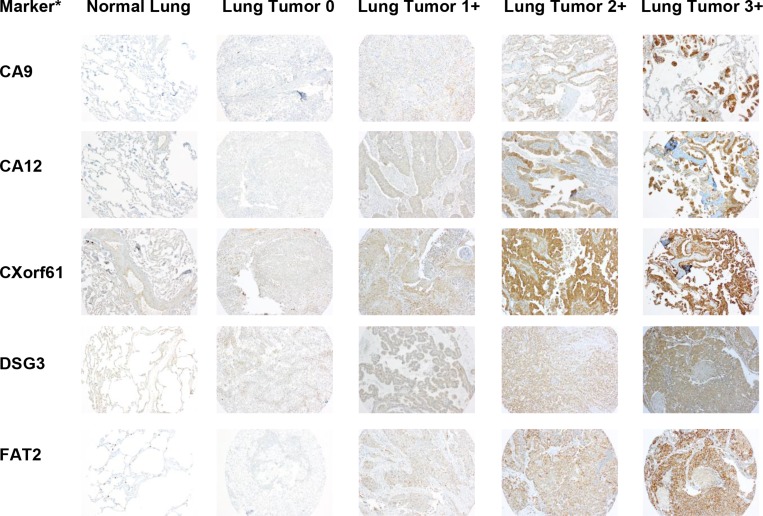
Representative images of IHC stained patient lung tumor and normal lung tissue specimens from the tissue microarray (TMA) for half of the selected markers A representative normal lung sample and representative lung tumor samples with scores of 0, 1+, 2+, and 3+ are shown for each marker. The images are taken at 10x magnification. ^*^Protein expression is stained but gene names are used to conserve space.

**Figure 4:**
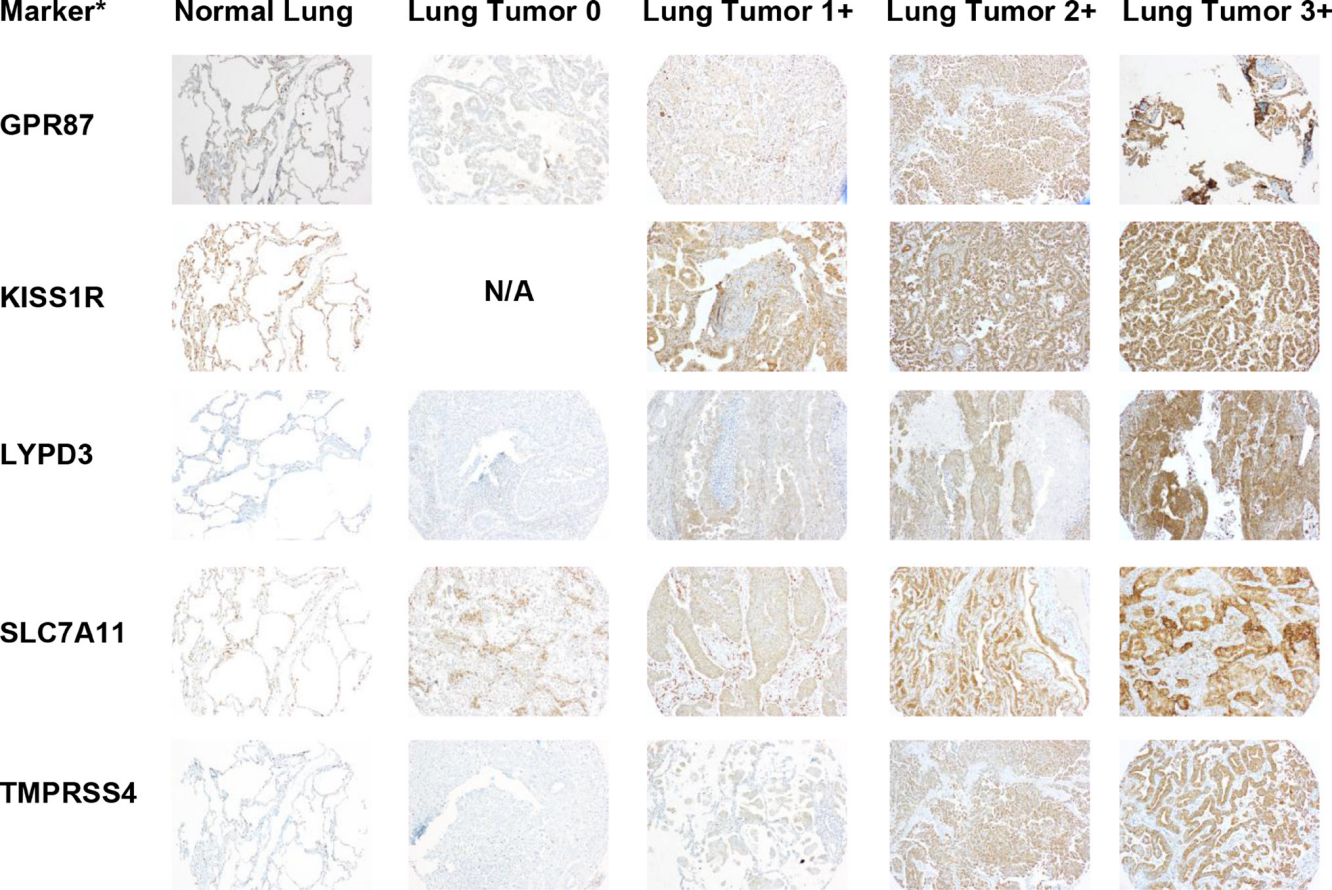
Representative images of IHC stained patient lung tumor and normal lung tissue specimens from the tissue microarray (TMA) for the remaining selected markers A representative normal lung sample and representative lung tumor samples with scores of 0, 1+, 2+, and 3+ are shown for each marker. The images are taken at 10x magnification. ^*^Protein expression is stained but gene names are used to conserve space.

Since mRNA expression does not always translate to protein, we needed to confirm protein expression of the selected markers. To do this, we performed immunohistochemistry (IHC) of a tissue microarray (TMA) consisting of lung tumor samples and adjacent normal lung samples, as well as several other control tissues (liver, spleen, and lymph node). Figures 3 and 4 show representative images of IHC-stained sections from lung tumors and non-neoplastic “normal” lung specimens from the TMA. As can be seen from the images, the lung tumor specimens have greater cell density compared to the normal lung specimens. Supplementary Figures 3 and 4 show higher magnification images of the lung tumor samples from the TMA.

The IHC staining was scored by a pathologist who specializes in thoracic oncology (F.K.K.) on a scale from 0 to 3+, with 3+ representing the strongest intensity. A summary of the scoring data for each marker in normal lung and lung tumor tissue is given in Tables 3 and 4. The IHC analysis of the other control tissues for each marker is given in Supplementary Table 32. Samples showing any percentage of cell staining were included in this analysis. For each of the markers, there were a percentage of tumor samples that had higher expression than the normal lung samples. The ten markers were divided into two groups based on expression in normal lung tissue (Tables 3 and 4). The first group consisted of six markers with limited (TMPRSS4) or no expression (CA12, FAT2, GPR87, LYPD3, and SLC7A11) in the normal lung samples (Table 3). For TMPRSS4, staining in normal lung was observed in only one of the eight specimens, and only 5% of the cells in that specimen had staining (Table 3). The remaining four markers (CA9, CXorf61, DSG3, and KISS1R) showed some expression in normal lung (Table 4). The only marker with high staining intensity (3+) in some (25%) of the normal lung specimens was CA9. For CXorf61, DSG3 and KISS1R, the expression was of low staining intensity (1+) for ≥50% of the normal lung specimens. The average percentage of cell staining is reported as a heterogeneity score (Tables 3 and 4 and Supplementary Table 32). When samples received a pathology score of 0, they also received a 100% heterogeneity score indicating that they were uniformly unstained. For samples that stained (pathology score of 1 or greater), the heterogeneity score indicates the percentage of cell staining regardless of intensity. Values close to 100% indicate more homogeneous staining. Some markers showed homogeneous staining, i.e. DSG3, KISS1R, and SLC7A11, whereas other markers were very heterogeneous, i.e. CA9 and LYPD3. For five of the markers, staining was observed in the lymphocytes in all tissues. The staining intensities of the lymphocytes were 3+ for GPR87, 2+ to 3+ for CXorf61, DSG3 and KISS1R, and 1+ to 2+ for FAT2. The TMA used in this study consists of a proportional representation of the histological subtypes of NSCLC as seen in the clinic. The markers were also analyzed for expression in the two predominant histological subtypes of NSCLC, adenocarcinoma and SCC (Tables 5 and 6). The remaining samples were of other histological classes (acinar cell carcinoma, adenosquamous carcinoma, large cell carcinoma, large cell neuroendocrine carcinoma, neuroendocrine carcinoma, mesothelioma or pleomorphic carcinoma), with a sample number ≤ 5 or were not otherwise specified, and these data were combined into a category termed as “other” due to the lower sample numbers.

**Table 3:** IHC scoring of marker expression in normal lung and lung cancer patient tissue samples: markers with limited or no expression in normal lung

Target^*^	Tissue type	Patient Tissue Samples (*n*)	Pathology Score					Heterogeneity Score^#^ (Average ± SD)
			0	1+	2+	3+	%≥1+	
**CA12**	**Normal Lung**	**8**	8	0	0	0	0%	100% ± 0%
	**Lung Tumor**	**97**	16	26	44	11	83%	84% ± 26%
**FAT2**	**Normal Lung**	**8**	8	0	0	0	0%	100% ± 0%
	**Lung Tumor**	**98**	5	35	39	19	95%	92% ± 17%
**GPR87**	**Normal Lung**	**8**	8	0	0	0	0%	100% ± 0%
	**Lung Tumor**	**100**	14	52	26	8	86%	90% ± 22%
**LYPD3**	**Normal Lung**	**8**	8	0	0	0	0%	100% ± 0%
	**Lung Tumor**	**97**	34	46	12	5	65%	61% ± 36%
**SLC7A11**	**Normal Lung**	**8**	8	0	0	0	0%	100% ± 0%
	**Lung Tumor**	**99**	14	59	23	3	86%	99% ± 5%
**TMPRSS4**	**Normal Lung**	**8**	7	0	1	0	12.5%	5%
	**Lung Tumor**	**100**	12	39	37	12	88%	83% ± 29%

^*^Protein expression is scored but gene names are used to conserve space.

^#^Heterogeneity score indicates the average percentage of cell staining in samples that stained regardless of pathology score. For samples with pathology scores of 0 only, 100% heterogeneity score indicates uniformly unstained.

**Table 4:** IHC scoring of marker expression in normal lung and lung cancer patient tissue samples: markers with some expression in normal lung

Target^*^	Tissue type	Patient Tissue Samples (*n*)	Pathology Score					Heterogeneity Score^#^ (Average ± SD)
			0	1+	2+	3+	%≥2+	
**CA9**	**Normal Lung**	**8**	4	2	0	2	25%	88% ± 13%
	**Lung Tumor**	**92**	13	24	26	29	60%	79% ± 32%
**CXorf61**	**Normal Lung**	**8**	4	4	0	0	0%	100% ± 0%
	**Lung Tumor**	**97**	12	35	45	5	52%	95% ± 17%
**DSG3**	**Normal Lung**	**8**	1	7	0	0	0%	100% ± 0%
	**Lung Tumor**	**95**	6	45	38	6	46%	99% ± 5%
**KISS1R**	**Normal Lung**	**8**	2	6	0	0	0%	100% ± 0%
	**Lung Tumor**	**96**	0	24	63	9	75%	100% ± 0%

^*^Protein expression is scored but gene names are used to conserve space.

^#^Heterogeneity score indicates the average percentage of cell staining in samples that stained regardless of pathology score. For samples with pathology scores of 0 only, 100% heterogeneity score indicates uniformly unstained.

**Table 5:** IHC scoring of marker expression in lung cancer patient tissue samples analyzed by lung cancer histology sub-type: markers with limited or no expression in normal lung

Target^*^	Tissue type	Patient Tissue Samples (*n*)	Pathology Score				
			0	1+	2+	3+	%≥1+
**CA12**	**Adenocarcinoma**	**61**	6	16	32	7	90%
	**SCC**	**10**	3	2	5	0	70%
	**Other**	**26**	7	8	7	4	73%
**FAT2**	**Adenocarcinoma**	**60**	2	17	27	14	97%
	**SCC**	**10**	1	7	1	1	90%
	**Other**	**28**	2	11	11	4	93%
**GPR87**	**Adenocarcinoma**	**62**	5	34	17	6	92%
	**SCC**	**11**	1	5	4	1	91%
	**Other**	**27**	8	13	5	1	70%
**LYPD3**	**Adenocarcinoma**	**61**	19	29	10	3	69%
	**SCC**	**10**	2	7	0	1	80%
	**Other**	**26**	13	10	2	1	50%
**SLC7A11**	**Adenocarcinoma**	**61**	8	36	15	2	87%
	**SCC**	**10**	1	6	2	1	90%
	**Other**	**28**	5	17	6	0	82%
**TMPRSS4**	**Adenocarcinoma**	**62**	5	25	25	7	92%
	**SCC**	**11**	1	4	4	2	91%
	**Other**	**28**	6	11	8	3	79%

^*^Protein expression is scored but gene names are used to conserve space.

**Table 6:** IHC scoring of marker expression in lung cancer patient tissue samples analyzed by lung cancer histology sub-type: markers with some expression in normal lung

Target^*^	Tissue type	Patient tissue samples (*n*)	Pathology score				
			0	1+	2+	3+	%≥2+
**CA9**	**Adenocarcinoma**	**56**	8	17	18	13	55%
	**SCC**	**10**	1	3	2	4	60%
	**Other**	**26**	4	4	6	12	69%
**CXorf61**	**Adenocarcinoma**	**60**	6	22	28	4	53%
	**SCC**	**10**	2	5	3	0	30%
	**Other**	**27**	4	8	14	1	56%
**DSG3**	**Adenocarcinoma**	**60**	4	25	27	4	52%
	**SCC**	**10**	1	5	3	1	40%
	**Other**	**25**	1	15	8	1	36%
**KISS1R**	**Adenocarcinoma**	**60**	0	14	40	6	77%
	**SCC**	**10**	0	5	4	1	50%
	**Other**	**26**	0	5	19	2	81%

^*^Protein expression is scored but gene names are used to conserve space.

### Marker expression in cell lines

**Figure 5:**
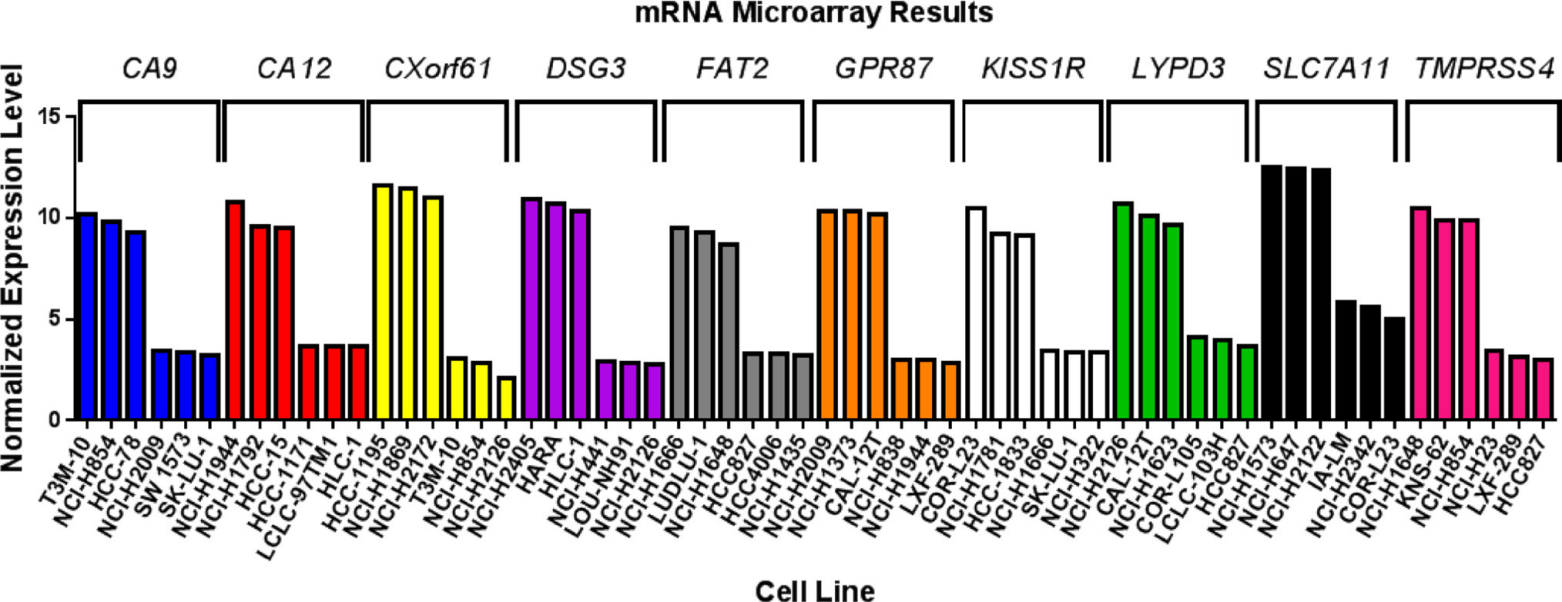
Marker expression in human lung cancer cell lines mRNA microarray data was analyzed for non-small cell lung cancer cell lines with high and low/no endogenous expression of each of the markers. The graph shows three cell lines with high and three cell lines with low/no expression for each marker.

As further confirmation, marker expression was evaluated in established human lung cancer cell lines. We have previously demonstrated that mRNA levels obtained from Affymetrix microarray data derived from cell lines are representative of levels obtained by quantitative real-time reverse-transcriptase polymerase chain reaction (qRT-PCR) of the same cell lines [30]. Hence, for each of the markers, we analyzed mRNA expression microarray data for cell lines (Supplementary Figures 5–14). For each marker, NSCLC cell lines with high and low/no mRNA expression were selected (Figure 5). These cell lines could be useful when developing models to test imaging or therapeutic agents targeting the markers.

### Survival analyses

**Figure 6:**
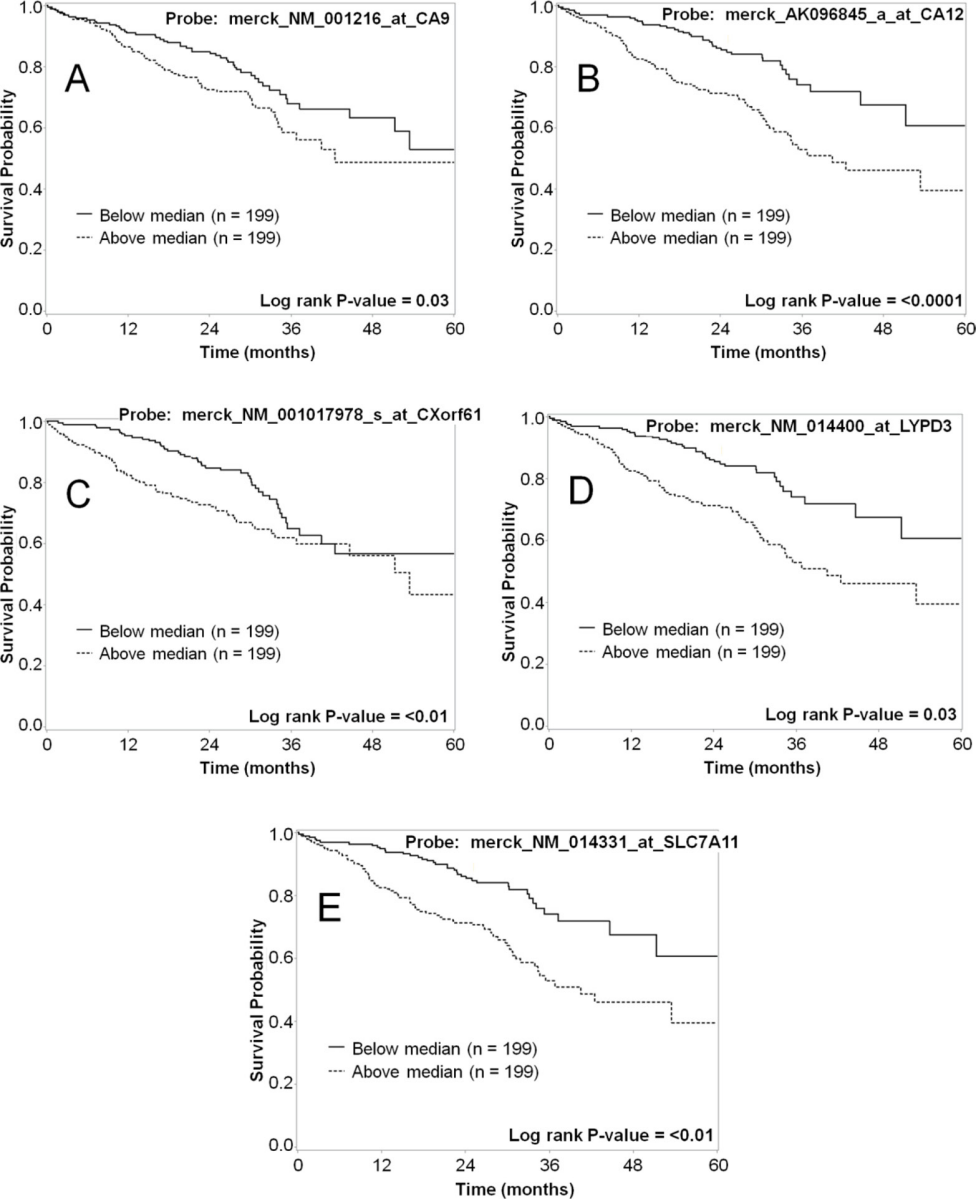
Representative Kaplan–Meier survival curves for lung cancer markers using mRNA expression data dichotomized based on the median-cut point The five-year survival for patients with high mRNA expression (dashed line) vs. low mRNA expression (solid line) was plotted for each of the markers. Shown are data for CA9 (**A**), CA12 (**B**), CXorf61 (**C**), LYPD3 (**D**), and SLC7A11 (**E**). Each of these markers shows a statistically significant difference in survival for patients with high vs. low mRNA expression.

**Figure 7:**
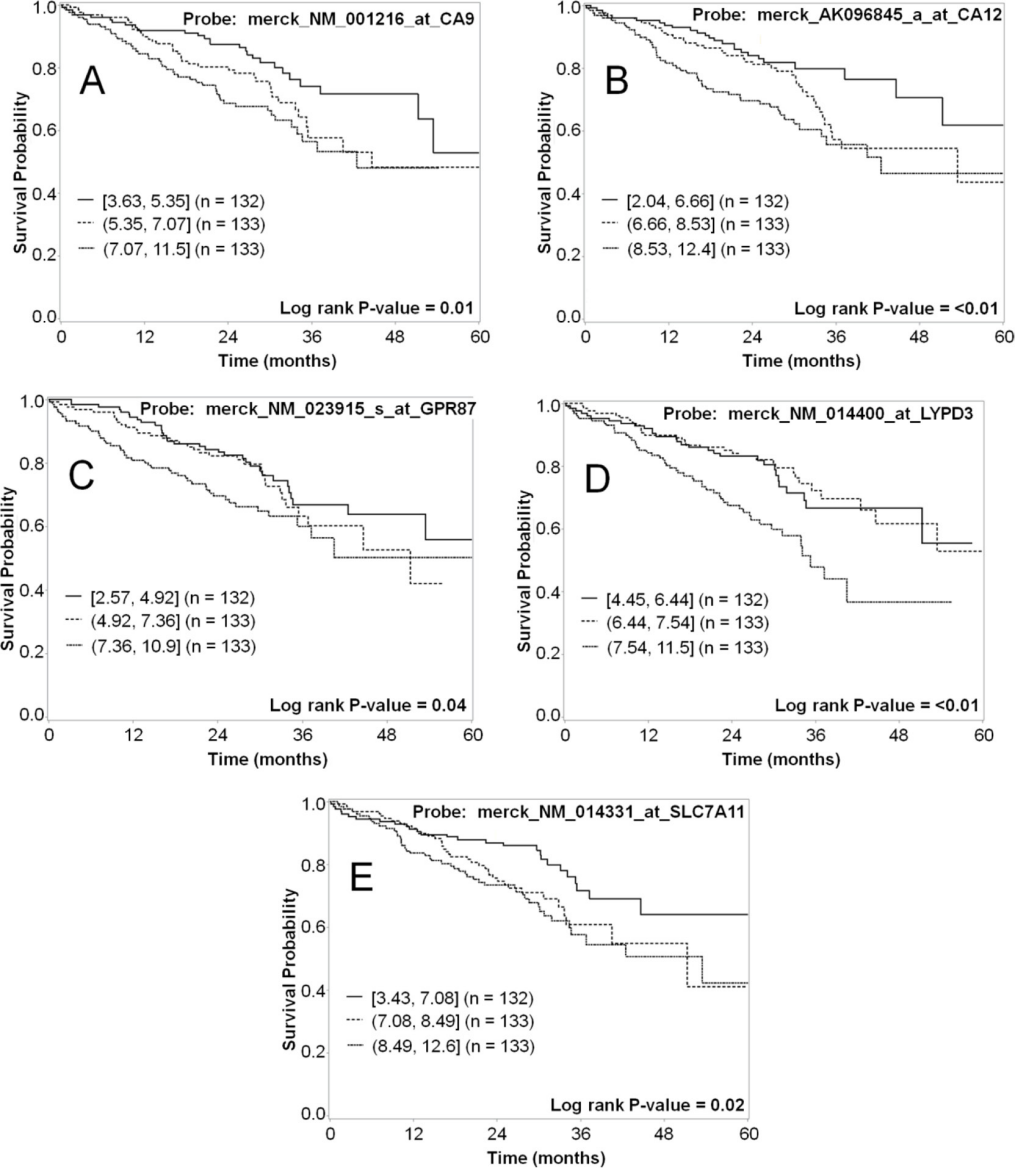
Representative Kaplan–Meier survival curves for lung cancer markers using mRNA expression data analyzed as tertiles The five-year survival for the third of patients with the highest mRNA expression (short dashed line); the middle third (dashed line); and the third with lowest expression (solid line) was plotted for each of the markers. Shown are data for CA9 (**A**), CA12 (**B**), GPR87 (**C**), LYPD3 (**D**), and SLC7A11 (**E**). Each of these markers shows a statistically significant difference in survival for patients with high vs. low mRNA expression.

As a further validation of each marker related to tumor biology and patient prognosis, both mRNA and protein expression data for the selected markers were evaluated in terms of patient survival. The mRNA expression was dichotomized at the median cut-point and the five-year survival was compared for the groups with high and low expression of the marker, and analyses were also conducted by tertile cutpoints (Table 7). High expression for five of the markers (CA9, CA12, CXorf61, LYPD3, and SLC7A11) significantly associated with worse survival (*p* < 0.05) when the data was dichotomized (Figure 6). For genes with multiple probesets (CA9 and CA12), the association was significant for all of the probesets (Table 7). When we analyzed the data using tertiles of expression, all of these markers were significantly associated with survival, except for CXorf61 (Table 7 and Figure 7). In addition, when analyzed by tertiles, GPR87 expression was significantly associated with survival (Table 7 and Figure 7C). The tertile analysis revealed that the third of specimens with highest LYPD3 expression was associated with worse survival relative to the two thirds of specimens with low expression values (Figure 7D), and for SLC7A11, two thirds of specimens with higher expression were associated with worse survival relative to the third with the lowest expression levels (Figure 7E).

**Table 7:** Significance of marker expression relative to survival by Affymetrix probe

Probe Name	Gene	Log Rank *P*-value for 2 groups (median split)	Log Rank *P*-value for 3 groups (tertile split)
merck_NM_001216_at_CA9	CA9	0.03^*^	0.01^*^
merck2_DQ892208_at_CA9	CA9	0.04^*^	0.02^*^
merck_NM_001218_s_at_CA12	CA12	<0.01^*^	0.02^*^
merck_AK096845_a_at_CA12	CA12	<0.0001^*^	<0.01^*^
merck2_BC087838_at_CA12	CA12	<0.01^*^	<0.01^*^
merck_NM_001017978_s_at_CXorf61	CXorf61	<0.01^*^	0.08
merck_BX538327_at_DSG3	DSG3	0.53	0.10
merck_NM_001944_a_at_DSG3	DSG3	0.33	0.65
merck2_M76482_at_DSG3	DSG3	0.48	0.88
merck_NM_001447_at_FAT2	FAT2	0.78	0.49
merck_NM_023915_s_at_GPR87	GPR87	0.15	0.04^*^
merck_NM_032551_s_at_KISS1R	KISS1R	0.94	0.88
merck_NM_014400_at_LYPD3	LYPD3	0.03^*^	<0.01^*^
merck_NM_014331_at_SLC7A11	SLC7A11	<0.01^*^	0.02^*^
merck_AI924527_a_at_TMPRSS4	TMPRSS4	0.75	0.46
merck2_NM_001083947_at_TMPRSS4	TMPRSS4	0.41	0.74
merck2_NM_183247_a_at_TMPRSS4	TMPRSS4	0.93	0.57

^*^p-values < 0.05 are considered significant and are emboldened.

**Figure 8:**
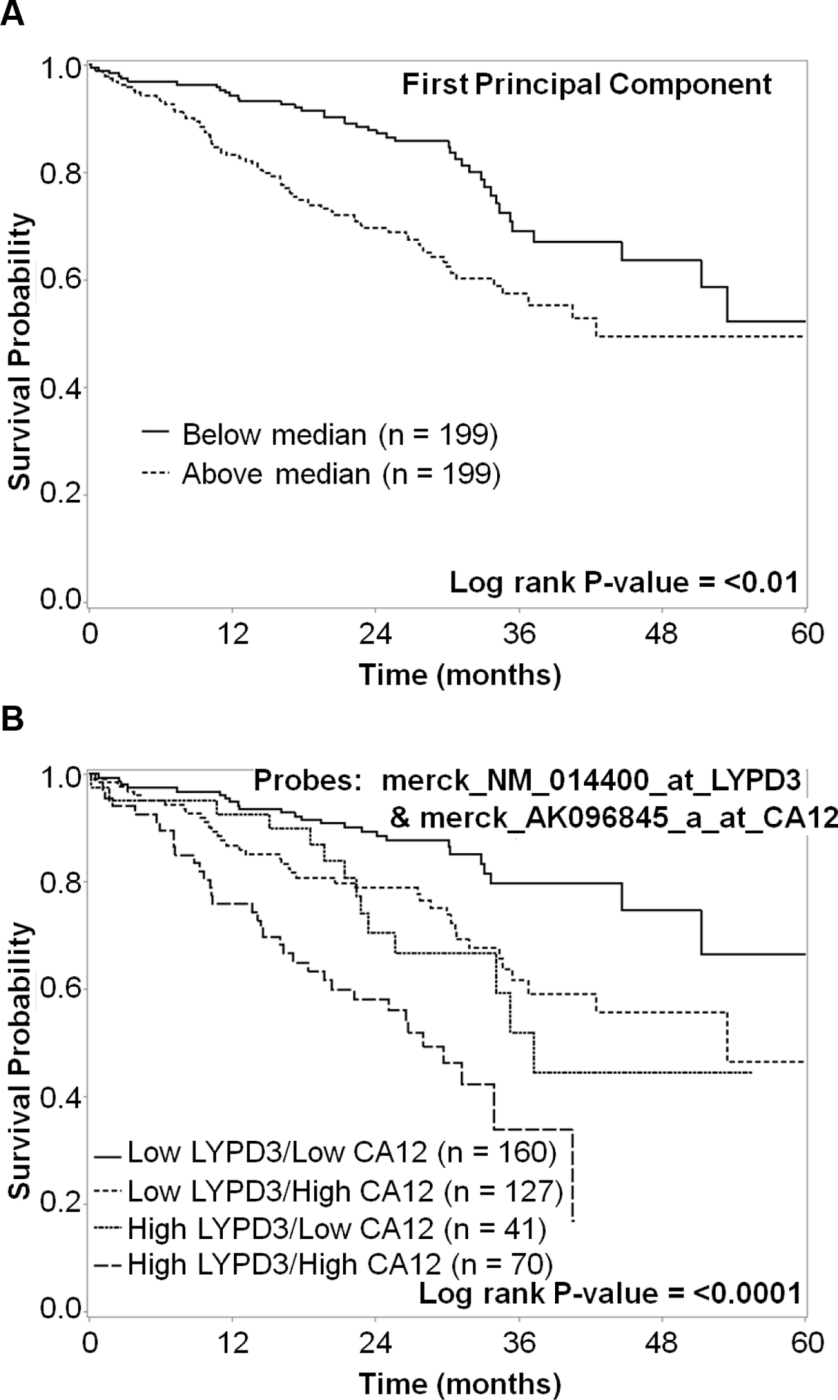
Kaplan–Meier survival curves for (**A**) metagene and (**B**) both LYPD3 and CA12 groupings. (A) For the metagene analysis, data were dichotomized based on the median cut-point. The five-year survival for patients with high metagene expression (dashed line) vs. low metagene expression (solid line) was plotted. There is a statistically significant difference in survival (*P* < 0.01). (B) For the LYPD3 and CA12 combined analysis, mRNA expression data were divided into four subgroups. The five-year survival for patients with low LYPD3/low CA12 (solid line), low LYPD3/high CA12 (dashed line), high LYPD3/low CA12 (short dashed line) and high LYPD3/high CA12 (long dashed line) was plotted. There is a statistically significant difference in survival (*P* < 0.0001).

A metagene signature was generated using the first principal component analysis (PCA) of the 8 probes that were significantly (*p* < 0.05) associated with survival based on the median split (three probes in CA12, two in CA9, CXorf61, LYPD3, and SLC7A11). The first principal component was dichotomized by the median into low and high expression. High expression of the metagene was significantly associated with worse survival (*P* < 0.01) (Figure 8A). Using a hierarchical analytical classification and regression tree (CART) approach on the same variables we had used for the PCA analysis, we determined LYPD3 and CA12 to be the two most predictive markers and determined their respective cut points. Four subgroups were identified (low LYPD3/low CA12, low LYPD3/high CA12, high LYPD3/low CA12 and high LYPD3/high CA12) and high expression of both markers was correlated with decreased survival, whereas low expression of both markers correlated with increased survival (*P* < 0.0001) (Figure 8B).

**Figure 9:**
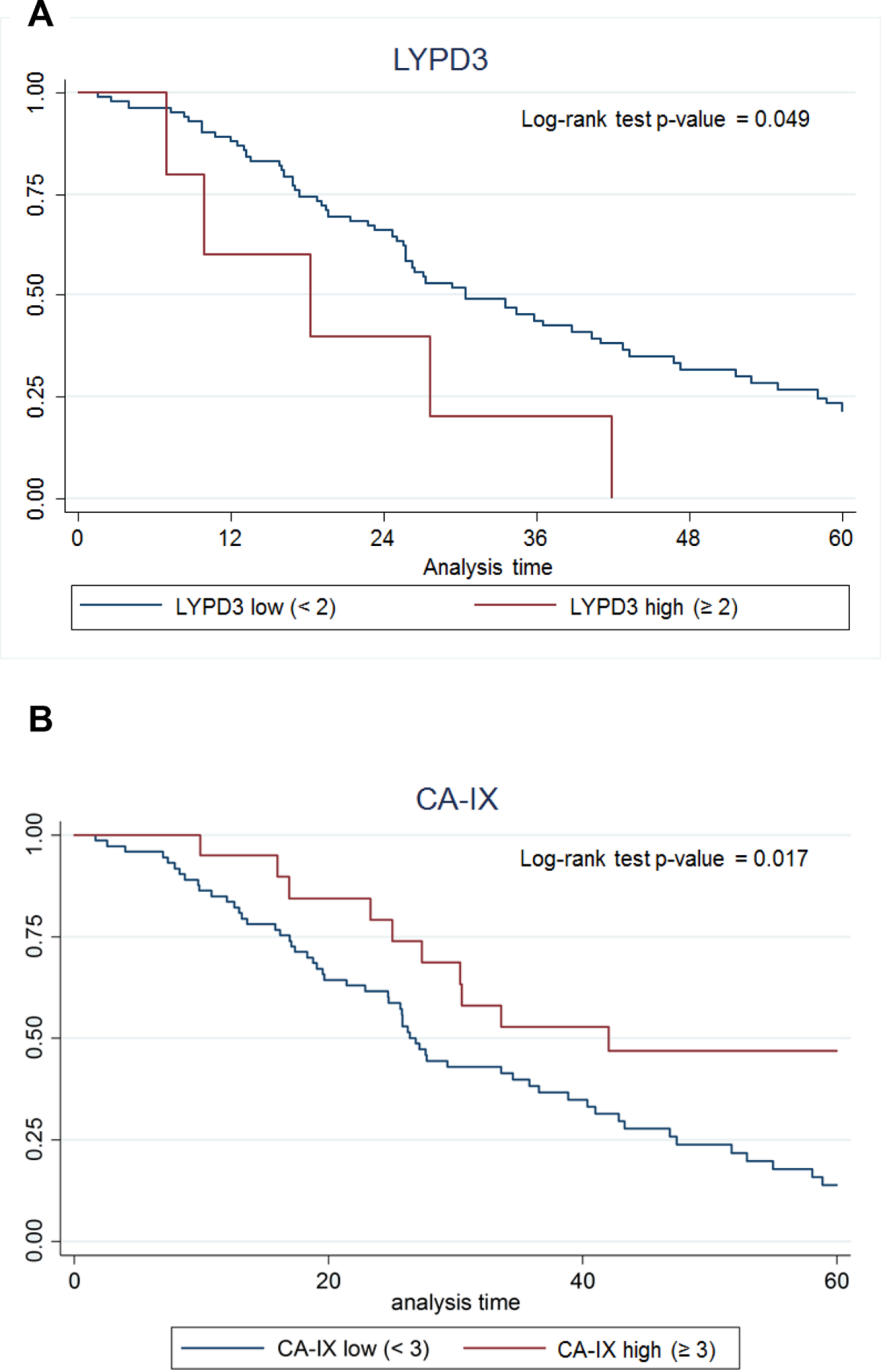
Kaplan–Meier survival curves for (**A**) LYPD3 and (**B**) CA-IX using IHC scoring data. The data were normalized by multiplying the staining intensity by the tumor cell staining percent. The five-year survival for patients with high protein expression (red line) vs. low protein expression (blue line) was plotted.

To analyze protein expression, we used both normalized and non-normalized data (described in the Methods section). We dichotomized the expression of the markers into a group with staining intensity ≥2+ and <2+ and assessed the survival for groups with high vs. low expression of each of the markers. The only marker for which high expression was significantly correlated with poor survival by this analysis was LYPD3 (Figure 9A). In a second analysis, four groupings were used (<1+, ≥1+ and <2+, ≥2+ and <3+, and ≥3+) and in this analysis CA-IX expression ≥3+ had significantly increased survival compared to CA-IX expression <3+ (Figure 9B).

## DISCUSSION

A major bottleneck in the development of targeted imaging and therapeutic agents for use in personalized medicine has been the availability of adequately vetted molecular targets. Individual targets are often reported in the literature for a given cancer type or clinical application, but it is rare that these target markers are compared with other potential targets simultaneously, using the same tissue specimens and analyses in order to estimate the potential utility of one marker relative to others. Potential targets are often reported based on mRNA expression alone, without confirmation of protein expression, which typically is the intended target. Additionally, elevated mRNA expression in cancer does not necessarily correspond to equivalent protein expression or subcellular localization. Tumor marker expression is often reported for only a small set of patient samples, only in tumor cell lines, or only reported for tumors without consideration of expression in surrounding normal tissues or normal tissues of concern for agent clearance or toxicity. Each of these concerns can lead to inadequately informed decisions about targets to pursue for development of targeted agents for a given application. To identify suitable targets for a given clinical application, studies are needed that can identify and compare marker expression among patient tumor sample sets that are representative of the intended target population and that include secondary levels of confirmation. Without adequate target discovery, costly decisions to undertake agent development may be made that are destined to fail.

Herein we report a systematic lung cancer cell-surface marker discovery effort. Our approach made use of the large amount of microarray data available for many clinical types of cancer. We specifically screened lung cancer array data for the high expression of genes in cancer samples that were poorly expressed in normal lung and several other key tissues. We then further narrowed the list to those genes we expected to be expressed at the cell surface. The goal of this work was to simultaneously identify and validate promising markers in lung cancer that can be used as targets for development of novel agents for use in personalized medicine. We focused on the identification of cell-surface markers because targeted agents that are designed for delivery of imaging contrast or cytotoxic payloads are typically conjugates with greater mass than small molecule drugs that can pass through the cell membrane via common transport mechanisms. By gene expression profiling of patient microarray data, we have identified greater than 200 putative cell-surface markers for lung cancer (Supplementary Table 1). From this list, we selected 10 promising markers (CA9, CA12, CXorf61, DSG3, FAT2, GPR87, KISS1R, LYPD3, SLC7A11, and TMPRSS4) for confirmation of protein expression in patient samples. By IHC, we determined differential protein expression of these markers in lung tumor specimens relative to normal lung and other normal tissues of concern for toxicity (Tables 3 and 4 and Supplementary Table 32). As secondary confirmations, we also demonstrated that lung cancer cell lines endogenously express these markers (Figure 5 and Supplementary Figures 5–14). These lung tumor cell lines can be useful for the development of agents targeted to these markers. We have also shown that survival correlates with expression for several of the described markers (Table 7 and Figures 6 and 7).

Many of the markers that were identified by this method had been previously reported for lung cancer or other cancer types (see below). This serves to validate our approach to discovery, but also highlights a key feature of our method; to identify and directly compare the relative utility of multiple markers simultaneously. Since the patient specimens used for identification and validation also have corresponding clinical data available, we were able to provide further evidence of the potential clinical relevance of each given marker, i.e. survival prognosis. Hence, we report a practical and systematic process that can be used to discover cell-surface markers that can be used for making decisions about targeted agent development for use in personalized medicine. This approach can also be applied to any class of cancer including rare cancer types that have not had the scrutiny of NSCLC.

Array data may miss many good targets as it is possible to have low mRNA expression with high corresponding protein levels. Other approaches such as proteomics and transcriptional sequencing may find other potential cancer markers. For example, proteomics approaches have been successfully applied toward the identification of membrane-associated proteins in lung tumor tissue relative to normal lung tissue [84, 85]. Nonetheless we have identified a suite of 10 potential cell-surface markers that identify the majority of lung tumor samples we analyzed.

Similar approaches have been used by other groups for lung cancer marker discovery. Nakamura *et al.* have several reports where a similar approach was used to identify lung cancer markers [86–90]. However, only one of these studies focused on cell-surface [86] and there are important differences, e.g. mRNA expression was determined by laser-capture microdissection of tumor cells, which decreases contamination from tumor infiltrating cells but also decreases the sample number that can be practically examined. RNA profiles are typically distorted by the processes required for laser capture microdissection limiting its usefulness in quantitative analyses. Nonetheless, useful markers can be identified in this way. Their cell-surface study identified SEZ6L2 as a cell-surface marker for lung cancer, which was also identified by our approach as having higher mRNA expression relative to normal tissues. However, it was not one of the candidates selected for further validation in our study due to its lower ranking based on marker score. Our initial screen identified 272 additional candidates that were not investigated further for this work, but still might prove useful. For example, SEZ6L2 was higher ranked than CA9 in our analysis but CA9 was selected due to the availability of molecular imaging probes targeting this marker and based on its potential applicability among several cancer types. Another similar study by Gugger *et al.* was limited to cell-surface G-protein-coupled receptor (GPCR) discovery and identified 5 GPCRs as being overexpressed [91]. Differential mRNA expression identified GPR87 as a lung SCC target, but protein expression was not confirmed [91]. Our current study effectively confirms the differential mRNA expression of GPR87 and goes on to demonstrate that protein expression was also higher in a large set of lung cancer samples. Recently, Botling *et al.*, used prognostic impact to select NSCLC biomarkers for IHC confirmation [92]. The study was not limited to cell-surface markers and the majority of markers discovered were intracellular, but the CADM1 cell-surface gene was identified and protein expression confirmed. Unfortunately, this (CADM1) protein has lower expression in tumor samples compared to normal lung samples [92]. We made this same observation in our mRNA expression microarray data set and, consequently, this protein was not selected for further study. In contrast to these studies, all of the markers selected for validation in our study had high protein expression in tumor cells compared to normal cells in a large fraction of the samples (Tables 3 and 4). These results suggest that using large sample numbers of whole tumor tissue digests is sufficient for the initial identification of markers that have high and broad expression among tumor cells and that laser-capture microdissection may be unnecessary for the detection of promising targets.

As stated above, a number of the markers in this study had previously been reported as expressed as mRNA or protein in lung cancer: CAIX [83, 93–107], CAXII [83, 108], KK-LC-1 [109–113], desmoglein 3 [114–116], GPR87 [91, 117–119], Ly6/PLAUR domain-containing protein 3 [118, 120–125] and solute carrier family 7 member 11 protein [63, 118]. However, we also confirmed protein expression in patient specimens for two novel lung cancer targets, i.e. FAT2 and KiSS-1R. Our results for KiSS-1R conflicted with previous reports that mRNA and protein levels of KISS1R were lower for NSCLC tissue relative to normal lung tissue [126, 127]. KISS1R levels were assessed by reverse-transcriptase polymerase chain reaction (RT-PCR) and Western Blot (WB). In addition, KISS1R expression was reported to be associated with better survival in patients with NSCLC [126]. In our study, we found higher mRNA and protein expression of KISS1R in lung tumors relative to normal lung tissue. Also, we did not observe an association between KISS1R mRNA expression in adenocarcinoma and improved survival. There are at least two mRNA splice variants resulting in different protein products. The different methodologies may be detecting different forms of the KISS1R product leading to these conflicting results. See Table 8 for a review of the literature regarding expression of all of the selected markers and comparison of results presented herein. Differences observed herein relative to published results are likely due to differences in the study populations and the way expression was evaluated.

**Table 8:** Literature review of the selected markers and comparison to results herein

Gene name	Protein name	Normal expression & function	Cancer expression & function	Comparison to results	References
CA9	Carbonic anhydrase IX (CAIX)	GI tract. Catalyzes the reversible hydration of CO_2_ to H_2_CO_3_.	↑ breast, lung, renal-cell cancers. ↑ SCC relative to other NSCLC. Correlates with poor survival, with a few exceptions.	General agreement.	[82, 83, 93–102, 105, 137–139]
CA12	Carbonic anhydrase XII (CAXII)	Brain, colon, rectum, esophagus, kidney, ovary, pancreas, prostate, testis and uterus. Catalyzes the reversible hydration of CO_2_ to H_2_CO_3_.	↑ breast, lung, renal-cell cancers. ↑ protein correlates with ↑ survival.	↓ survival with ↑ mRNA.	[82, 83, 108, 140–143]
CXorf61	Kita-kyushu lung cancer antigen 1 (KK-LC-1)	Testis. Cancer testis antigen family.	↑ mRNA in NSCLC. No correlation with survival.	mRNA results were not significant for SCC. ↑ mRNA correlates with ↓ survival. First report of ↑ protein in lung adenocarcinoma.	[109–113]
DSG3	Desmoglein 3	Normal stratified squamous epithelia, GI tract. Cadherin superfamily.	↑ SCCs, ↓ adenocarcinoma. ↑ increased survival. ↓ higher tumor grade.	↑ SCCs, ↑ adenocarcinoma. No correlation with survival.	[114–116, 144–147]
FAT2	Protocadherin Fat 2	Cerebellum, epidermis. Cadherin superfamily.	↑ esophageal, gastric, head and neck, ovarian, pancreatic cancers, cutaneous SCC and NSCLC. ↑ poor NSCLC survival.	No correlation with survival.	[148–152]
GPR87	G-protein coupled receptor 87	Prostate, placenta, head and neck. P2Y purin receptor family.	↑ bladder cancer and SCC of lung, cervix, head and neck, skin. Not elevated in adenocarcinoma. ↑ mRNA ↓ survival.	General agreement except ↑ in both SCC and adenocarcinoma.	[91, 117–119, 153, 154]
KISS1R	Kiss-1R	Placenta, pancreas, pituitary gland, brain. Role in normal and pathologic physiology, reproduction and pubertal development, hypothalamic-pituitary-gonadal axis.	↑ bladder, hepatocellular, ovarian, pancreatic, renal cell, thyroid cancers. ↓ endometrial, esophageal, NSCLC and prostate cancers. ↑ NSCLC survival.	↑ NSCLC and no correlation with survival.	[71, 73–76, 78, 126, 127, 155–170]
LYPD3	Ly6/PLAUR domain-containing protein 3	Squamous epithelia, placenta and peripheral blood leukocytes.	↑ breast, colorectal, gastric, lung, melanoma and urothelial cancers. Correlates with poor NSCLC survival.	General agreement.	[118, 120–125, 171–180]
SLC7A11	Cystine/glutamate transporter (xCT)	Role in regulation of oxidative stress and maintenance of the cysteine-cystine redox cycle. Brain, spinal cord and pancreas.	↑ NSCLC and many cancer types. Role in drug resistance.	Confirmed previous results in NSCLC with a larger data set and determined association with ↓ survival.	[62, 63, 181–187]
TMPRSS4	Transmembrane protease serine 4 protein	GI tract, urogenital tract, eye and skin.	↑ breast, cervical, colorectal, gallbladder, gastric, liver, lung, ovarian, pancreatic and thyroid cancers. Cell invasion, migration and adhesion. ↑ correlates with poor survival.	No correlation with survival.	[188–207]

Five of the selected markers have either known imaging agents (CA9, CA12, KISS1R and SLC7A11) or known high affinity ligands (KISS1R) or inhibitors (TMPRSS4) and structure activity relationships (SAR) for development of imaging agents. We have recently developed monoclonal antibody agents against both CA9 and CA12 [25], there are numerous reports of CA9 targeted imaging agents [35–58], and there is a commercially available near-infrared (NIR) fluorescent carbonic anhydrase inhibitor-based agent (Hypoxisense, PerkinElmer). Two ^18^F-glutamate derivative PET agents and an ^18^F-aminosuberic acid derivative PET agent have also been developed that target the x_C_^-^ transporter (SLC7A11) [62–69]. Recently, a fluorescent cystine derivative has also been developed for imaging the x_C_^-^ transporter (SLC7A11) [70]. High-affinity agonist (including metastin analogs and fluorobenzoyl pentapeptides) [71–75, 79] and antagonist (including 2-acylamino-4,6-diphenyl-pyridine derivatives) [76–78] ligands are known for the KiSS-1 receptor (KISS1R). Recently, fluorescently labeled ligands have been developed for studying KISS1R [59–61]. In addition, a series of 2-hydroxydiarylamide derivatives have been reported as potential TMPRSS4 serine protease inhibitors [80]. These targeting moieties could be used to develop novel targeted imaging agents against these markers. For the remaining markers which have no known ligands or SAR (CXorf61, DSG3, FAT2, GPR87, and LYPD3), targeting moieties can be developed using various approaches, such as humanized monoclonal antibodies or antibody fragments, or phage display and one-bead one-compound (OBOC) combinatorial library screens. High mRNA expression of six of the NSCLC markers (CA9, CA12, CXorf61, GPR87, LYPD3 and SLC7A11) correlated with decreased patient survival (Table 7, and Figures 6 and 7). Hence, these cell-surface markers have potential for development of non-invasive diagnostic imaging agents that could provide additional information on patient prognosis.

Six of the markers studied are potential targets for development of smart-bomb or Trojan horse therapies that deliver cytotoxic agents or therapeutic radionuclides specifically to cancer cells. CXorf61, DSG3, GPR87, KISS1R, LYPD3, and SLC7A11 genes had high mRNA expression in patient lung tumor specimens and low mRNA expression in tissues of concern for toxicity (Table 1, Supplementary Tables 4, 5 and 7–10, and Figure 1B–1D, and Supplementary Figure 1B, 1C and 1E). Although DSG3 and LYPD3 are expressed in epithelial layers, and KISS1R and SLC7A11 are expressed in normal brain, the basement membrane and blood-brain barrier would likely inhibit uptake in those normal tissues. A recent report describes an antibody-drug conjugate targeting LYPD3 which showed efficacy in preclinical mouse models of lung cancer and is currently being tested in clinical trials [125]. IHC staining revealed that GPR87, LYPD3, and SLC7A11 had high positivity in lung tumor tissues but did not stain normal lung tissue (Table 3). High mRNA expression of four of these markers, CXorf61, GPR87, LYPD3 and SLC7A11, significantly correlated with decreased survival (Table 7, Figures 6C-6E and 7C-7E) and high IHC staining also correlated with decreased survival for LYPD3 (Figure 9A), indicating the potential need for improved therapies for these patients.

When combining mRNA expression data for LYPD3 and CA12 we can identify four subgroups (low LYPD3/low CA12, low LYPD3/high CA12, high LYPD3/low CA12 and high LYPD3/high CA12). High expression of both markers was correlated with decreased survival, whereas low expression of both markers correlated with increased survival (Figure 8B). These results could help guide treatment plans for patients with expression of these markers. In addition, a bivalent targeting ligand with low affinity for each individual target, but high affinity for tumor cells expressing both markers, would focus treatment on tumors with the worst prognosis, but spare normal tissues that express only one of the targets and decrease unwanted systemic toxicities [128]. This bivalent targeting ligand could be used as both a therapeutic agent and companion diagnostic.

We report a systematic approach for the identification of novel lung cancer markers with cell-surface expression that may have potential utility in personalized medicine applications. Our approach supported existing literature describing a number of known markers overexpressed in lung cancers and further showed whether or not expression of these markers significantly correlates with prognosis. The large numbers of patient lung tumor and normal tissue specimens included in the analysis enabled the discrimination of tumor expression from normal lung tissue as well as the analysis of recognized sub-types of NSCLC. Evaluation of both mRNA and protein expression results allows for comparison of the two major molecular manifestations of gene expression and the confirmation of cell-surface markers targetable for molecular imaging and delivery of cytotoxic agents or radionuclides. Inclusion of clinical data with corresponding mRNA and protein expression allowed for the correlation of marker expression with prognosis. Evaluation of a set of promising markers using the same tissue and data sets allows for the simultaneous evaluation of the relative utility of each marker as a target for specific clinical applications. Determination of marker expression in established lung cancer cell lines provides laboratory tools for the development of novel agents that target these specific markers. We identified 208 potential cell-surface markers specifically overexpressed in some lung tumors, but not in normal lung. We further demonstrated that 10 of these targets were detectable by immunohistochemistry and therefore good candidates for the development of novel targeted therapeutics. Some of our candidates are already being targeted in this way. For example, the xCT transporter (SLC7A11) was confirmed to be a potentially robust lung tumor imaging marker and PET imaging agents are already being developed for this transporter but have yet to be applied toward use in lung tumor imaging, except in a small pilot clinical trial [62–70]. Additionally, the Ly6/PLAUR domain-containing protein 3 (LYPD3) emerged as a novel target for development of a lung cancer targeted therapy, which could be co-developed with a companion imaging agent for the personalized treatment of lung cancer.

## MATERIALS AND METHODS

### Expression (mRNA) profiling of microarray data

#### Tissue data and analyses

Compilation and quality control assessments of public mRNA expression microarray data sets were carried out in the Moffitt Biomedical Informatics and Molecular Genomics Laboratories. Many separate GEO [129] datasets were compiled that included Affymetrix mRNA expression array data from lung tumor (LT), lung normal (LN), and non-lung normal (NLN) patient tissue specimen cohorts, consisting of 262, 161, and 246 samples, respectively (Supplementary Tables 33 and 34). The datasets were combined and normalized together. IRON [130] was used to normalize all samples against the median sample (GSM475685). Affymetrix probesets that do not detect cataloged human genes were removed prior to further analysis. The list of genes evaluated were further filtered using a curated list of probesets (Supplementary Table 35) that correspond to only secreted or outer membrane proteins as derived from manual assessment and Gene Ontology terms [131].

For the remaining probesets, averages (*avg*) and standard deviations (*sd*) of log_2_ intensities were calculated within lung tumors and normals, separately. A cutoff of avg_normal_ +3 sd_normal_ was used for determining elevated expression in lung tumor samples for each probeset. Percentages of samples with elevated expression were calculated within lung tumors (*% elevated*_*tumor*_) and normals (*% elevated*_*normal*_), separately. Log_2_ ratios (*avg*_*tumor_elevated*_ *– avg*_*normal*_) of average elevated tumor (samples above the +3 sd_normal_ cutoff) vs. average normal, two-sided T-tests and Mann-Whitney U-tests, and Hellinger distances were calculated between the lung tumor and normal groups. Probesets were identified as elevated in lung tumors using the following criteria: *avg*_*tumor_elevated*_ > 5, *% elevated*_*tumor*_ > 25%, *log*_*2*_ *ratio elevated* ≥ 2 (4-fold), both lung all-tumor vs. all-normal T-test and U-tests < 4.2237e-6 (Bonferroni correction for P/N = 0.05/11,838), and Hellinger distance > 1/3^rd^. Elevated genes were then ranked in decreasing order by a MarkerScore, calculated as (*% elevated*_*tumor*_ – *% elevated*_*normal*_) * (*log*_*2*_ *ratio elevated*). These genes were then manually assessed for cell-surface/membrane location using UniProt and the Human Protein Atlas and the gene was kept in the analysis if either source listed the protein as cell-surface/membrane. The Marker Score was used to rank genes in priority for additional manual inspection (including identifying probesets with high intensity and broad expression in lung tumors relative to normal lung and minimal expression outside of lung) and experimental validation as described below.

### Cell line data and analyses

Additional verification of lung tumor expression was assessed using non-small cell lung cancer (NSCLC) cell line gene expression data from the Cancer Cell Line Encyclopedia (CCLE) [132]. All 991 CEL files were normalized using IRON [130] against the median sample. Principal component analysis (PCA) was performed, and samples identified that did not cluster with other samples of the same conformed site of origin (SOO). For 51 of these samples, literature and other notations in the metadata were used to support reclassification of the originally reported SOO to a new conformed SOO that agreed with the gene expression metadata. Twenty outlier samples, for which no justification could be found for altering their reported SOO, were discarded due to large disagreement between gene expression and reported SOO. These remaining 971 samples were then de-batched using COMBAT [133], using the batch reported in the metadata, and conformed SOO as covariate. From this batch-corrected data set, 114 cell lines were identified as NSCLC.

### Immunohistochemistry (IHC) of tissue microarray (TMA)

An existing lung cancer tissue microarray (TMA), constructed by the Moffitt Tissue Core from formalin-fixed and paraffin-embedded (FFPE) samples, was utilized. The patient demographics for this TMA are provided in Supplementary Table 36. The TMA contains cores from 106 lung tumor samples, 8 normal lung samples, 4 liver samples, 6 spleen samples, and 2 lymph node samples. The TMA consists of cylindrical punches of the FFPE blocks using a Manual Tissue Arrayer (Beecher Instruments). The tumor samples on the TMA are initial biopsy samples that correspond to pathologies of all stages. However the TMA was retrospectively constructed only using tissues from patients that eventually reached Stage IV disease. Primary antibody optimizations were carried out by titrating antibodies at various dilutions on control tissues recommended by the manufacturer (Supplementary Table 37). Slides were stained using a Ventana Discovery XT automated system (Ventana Medical Systems, Tucson) as per the manufacturer’s protocol using proprietary reagents. Slides were deparaffinized on the automated system with EZ Prep solution (Ventana). Heat-induced antigen retrieval methods were used in either RiboCC or Cell Conditioning 1 (Ventana) as listed in Supplementary Table 37. Primary antibodies were diluted using Dako diluent (Carpenteria, CA, USA) at the optimal ratios and incubation times listed in Supplementary Table 37. The appropriate anti-mouse or anti-rabbit secondary antibody (Ventana Omnimap or Ultramap) was used for 12 to 20 min incubation. The Ventana ChromoMap kit detection system was used first and then slides were counterstained with hematoxylin. Following staining, slides were dehydrated and coverslipped. Positive controls were used following the antibody manufacturer recommendations. Negative controls were established by omitting the antibodies during the primary antibody incubation step.

Slides were scored by a pulmonary pathologist (F.K.K.) and each sample given a numerical intensity score (0–3) where 0 = negative, 1 = weak, 2 = moderate and 3 = strong staining. The percentage of tumor cell staining was also scored. This percentage is independent of the staining intensity. A heterogeneity score was calculated by determining the average cell staining percent for cells that stained, regardless of pathology score. For samples with pathology scores of 0 only, a 100% heterogeneity score indicates uniformly unstained.

### Lung cancer patients and patient data

The protocol for this study was approved by the University of South Florida Institutional Review Board. The study included 442 lung cancer patients that were diagnosed with adenocarcinoma and recruited from Moffitt Cancer Center’s Total Cancer Care (TCC^™^) program [134] between April 2006 and August 2010. Patients for this analysis provided informed consent to the TCC^™^ protocol either at Moffitt (No. = 186) or one of eighteen TCC^™^ consortium/affiliate institutions (No. = 282). The demographic information of the patient cohort and details of the study design have been published elsewhere [135].

### Statistical analyses

GraphPad Prism (Version 5.04, La Jolla, CA, USA) was used to generate the box/whiskers plots. Box plot whiskers represent the minimum to maximum values in the group, the box represents the 50th percentile, and the center line represents the median value. SAS software (Version 9.4, Cary, NC, USA) was used for data analysis. Dunnett’s multiple comparison was used for testing lung tumor (control) versus normal tissues and for testing normal lung (control) to different lung cancer histologies. Tukey’s all pairwise comparisons were used for testing between different cancer histologies. For all tests, *p* ≤ 0.05 was considered significant.

Statistical analyses were performed using Stata/MP 12.1 (StataCorp LP, College Station, TX, USA). Survival analyses were performed using Kaplan–Meier survival curves and the log-rank test. Overall survival was the primary endpoint and was assessed from the date of surgery to the date of last follow-up or death. Among individuals without an event (i.e., death), censoring occurred at either 5 years or date of last follow-up if less than 5 years. Normalized IHC values were calculated by taking the product of the staining intensity and percent tumor cell staining for each marker. Principal component analysis (PCA) was utilized to generate a “metagene” score of mRNA gene probes. We utilized a classification and regression tree (CART) approach to explore potential novel biomarker combinations. CART is a nonparametric data-mining tool that can segment data into meaningful subgroups and has been adapted for failure time data [136] using the Martingale Residuals of a Cox model to approximate chi-square values for any number of biomarker combinations.

## SUPPLEMENTARY MATERIALS FIGURES AND TABLES











## Abbreviations

ALK: anaplastic lymphoma kinase
Avg: average
CART: classification and regression tree
CCLE: Cancer Cell Line Encyclopedia
CT: computed tomography
EGFR: epidermal growth factor receptor
^18^F-FDG: ^18^F-fluorodeoxyglucose
FFPE: formalin-fixed and paraffin-embedded
GPCR: G-protein-coupled receptor
IHC: immunohistochemistry
LDCT: low-dose computed tomography
LN: lung normal
LT: lung tumor
MRI: magnetic resonance imaging
NIR: near: infrared
NLN: non-lung normal
NLST: National Lung Screening Trial
NSCLC: non-small cell lung cancer
OBOC: one-bead one-compound
PCA: principal component analysis
PCR: polymerase chain reaction
PET: positron emission tomography
qRT-PCR: quantitative real-time reverse-transcriptase polymerase chain reaction
RT-PCR: reverse-transcriptase polymerase chain reaction
SAR: structure activity relationships
SCC: squamous cell carcinoma
Sd: standard deviation
SOO: site of origin
TCC: Total Cancer Care
TMA: tissue microarray
WB: Western blot
