# Supplementary material for: Cell-surface marker discovery for lung cancer
Source: Oncotarget. 2017 Dec 7;8(69):113373–402. doi: 10.18632/oncotarget.23009 (PMC5768334; doi:10.18632/oncotarget.23009)
Supplement: Supplementary file 1 [file oncotarget-08-113373-s001.pdf]

Cell-surface marker discovery for lung cancer

SUPPLEMENTARY MATERIALS

Supplementary Table 1: List of potential surface accessible probesets significantly elevated in tumors. See Supplementary_Table_1

Supplementary Table 2: Summary of mRNA data for *CA9* from Figure 1

Tissue type	*N*	Mean	SD	Adjusted *p* value*
Lung Tumor	262	5.78	1.26	_
Normal Lung	161	4.82	0.49	**<0.0001**
Adrenal Gland	36	4.72	0.33	**<0.0001**
Heart	10	6.11	0.77	0.88
Kidney	9	5.28	0.32	0.53
Liver	91	5.42	0.68	**0.0080**
Lymph Node	56	4.27	0.46	**<0.0001**
Small Intestine	6	7.10	1.36	**0.0039**

*Adjusted *p* values were calculated by Dunnett's multiple comparisons for lung tumor (control) versus normal tissues.

Supplementary Table 3: Summary of mRNA data for *CA12* from Figure 1

Tissue type	*N*	Mean	SD	Adjusted *p* value*
Lung Tumor	262	5.91	1.66	_
Normal Lung	161	4.23	0.55	**<0.0001**
Adrenal Gland	36	3.80	0.18	**<0.0001**
Heart	10	4.08	1.04	**<0.0001**
Kidney	9	10.25	0.50	**<0.0001**
Liver	91	3.82	0.33	**<0.0001**
Lymph Node	56	3.74	0.30	**<0.0001**
Small Intestine	6	4.07	0.59	**0.0007**

*Adjusted *p* values were calculated by Dunnett's multiple comparisons for lung tumor (control) versus normal tissue

Supplementary Table 4: Summary of mRNA data for *CXorf61* from Figure 1

Tissue type	*N*	Mean	SD	Adjusted *p* value*
Lung Tumor	262	4.39	1.99	_
Normal Lung	161	3.10	0.27	**<0.0001**
Adrenal Gland	36	3.35	0.20	**<0.0001**
Heart	10	3.07	0.11	**0.0118**
Kidney	9	2.98	0.07	**0.0094**
Liver	91	3.21	0.20	**<0.0001**
Lymph Node	56	3.08	0.07	**<0.0001**
Small Intestine	6	3.14	0.08	0.13

*Adjusted *p* values were calculated by Dunnett's multiple comparisons for lung tumor (control) versus normal tissues.

Supplementary Table 5: Summary of mRNA data for *DSG3* from Figure 1

Tissue type	*N*	Mean	SD	Adjusted *p* value[*]
Lung Tumor	262	4.92	2.94	_
Normal Lung	161	2.90	0.28	**<0.0001**
Adrenal Gland	36	2.83	0.15	**<0.0001**
Heart	10	3.04	0.15	**0.0161**
Kidney	9	2.91	0.06	**0.0136**
Liver	91	2.92	0.13	**<0.0001**
Lymph Node	56	2.83	0.08	**<0.0001**
Small Intestine	6	3.09	0.24	0.13

[*]Adjusted *p* values were calculated by Dunnett's multiple comparisons for lung tumor (control) versus normal tissues.

Supplementary Table 6: Summary of mRNA data for *FAT2* from Figure 1

Tissue type	*N*	Mean	SD	Adjusted *p* value[*]
Lung Tumor	262	4.69	1.47	_
Normal Lung	161	3.80	0.24	**<0.0001**
Adrenal Gland	36	4.16	0.30	**0.0170**
Heart	10	3.77	0.15	**0.0239**
Kidney	9	3.92	0.19	0.13
Liver	91	3.80	0.26	**<0.0001**
Lymph Node	56	3.67	0.28	**<0.0001**
Small Intestine	6	3.81	0.19	0.18

[*]Adjusted *p* values were calculated by Dunnett's multiple comparisons for lung tumor (control) versus normal tissues.

Supplementary Table 7: Summary of mRNA data for *GPR87* from Figure 1

Tissue type	*N*	Mean	SD	Adjusted *p* value[*]
Lung Tumor	262	5.81	2.40	_
Normal Lung	161	3.16	0.41	**<0.0001**
Adrenal Gland	36	3.02	0.10	**<0.0001**
Heart	10	3.05	0.07	**<0.0001**
Kidney	9	2.99	0.11	**<0.0001**
Liver	91	2.98	0.13	**<0.0001**
Lymph Node	56	3.07	0.14	**<0.0001**
Small Intestine	6	2.98	0.06	**0.0001**

[*]Adjusted *p* values were calculated by Dunnett's multiple comparisons for lung tumor (control) versus normal tissues.

Supplementary Table 8: Summary of mRNA data for *KISS1R* from Figure 1

Tissue type	*N*	Mean	SD	Adjusted *p* value*
Lung Tumor	262	5.44	1.48	_
Normal Lung	161	3.54	0.33	**<0.0001**
Adrenal Gland	36	3.16	0.14	**<0.0001**
Heart	10	3.22	0.08	**<0.0001**
Kidney	9	3.19	0.13	**<0.0001**
Liver	91	3.31	0.19	**<0.0001**
Lymph Node	56	4.06	0.41	**<0.0001**
Small Intestine	6	3.50	0.17	**<0.0001**

*Adjusted *p* values were calculated by Dunnett's multiple comparisons for lung tumor (control) versus normal tissues.

Supplementary Table 9: Summary of mRNA data for *LYPD3* from Figure 1

Tissue type	*N*	Mean	SD	Adjusted *p* value*
Lung Tumor	262	6.37	1.44	_
Normal Lung	161	5.12	0.33	**<0.0001**
Adrenal Gland	36	5.97	0.62	0.15
Heart	10	6.05	0.40	0.92
Kidney	9	6.30	0.32	>0.99
Liver	91	5.33	0.57	**<0.0001**
Lymph Node	56	6.24	0.33	0.96
Small Intestine	6	5.90	0.52	0.85

*Adjusted *p* values were calculated by Dunnett's multiple comparisons for lung tumor (control) versus normal tissues.

Supplementary Table 10: Summary of mRNA data for *SLC7A11* from Figure 1

Tissue type	*N*	Mean	SD	Adjusted *p* value*
Lung Tumor	262	6.51	2.08	_
Normal Lung	161	4.14	0.79	**<0.0001**
Adrenal Gland	36	3.08	0.55	**<0.0001**
Heart	10	3.32	0.63	**<0.0001**
Kidney	9	2.91	0.06	**<0.0001**
Liver	91	3.11	0.25	**<0.0001**
Lymph Node	56	3.77	0.46	**<0.0001**
Small Intestine	6	3.15	0.19	**<0.0001**

*Adjusted *p* values were calculated by Dunnett's multiple comparisons for lung tumor (control) versus normal tissues.

Supplementary Table 11: Summary of mRNA data for *TMPRSS4* from Figure 1

Tissue type	*N*	Mean	SD	Adjusted *p* value*
Lung Tumor	262	7.30	1.61	_
Normal Lung	161	4.68	0.51	**<0.0001**
Adrenal Gland	36	4.07	0.34	**<0.0001**
Heart	10	4.41	0.35	**<0.0001**
Kidney	9	5.62	0.79	**<0.0001**
Liver	91	4.40	0.26	**<0.0001**
Lymph Node	56	4.02	0.28	**<0.0001**
Small Intestine	6	6.25	1.67	0.14

*Adjusted *p* values were calculated by Dunnett's multiple comparisons for lung tumor (control) versus normal tissues.

Supplementary Table 12: Summary of mRNA data for *CA9* from Figure 2

Cancer type	*N*	Mean	SD	Adjusted *p* value*
Normal Lung	161	4.82	0.49	_
Squamous Cell Carcinoma	46	6.23	1.33	**<0.0001**
Adenocarcinoma	150	5.46	1.09	**<0.0001**
Large Cell Carcinoma	18	5.81	1.40	**0.0003**
Other	48	6.36	1.34	**<0.0001**

*Adjusted *p* values were calculated by Dunnett's multiple comparisons for normal lung (control) versus lung cancer histologies.

Supplementary Table 13: Summary of mRNA data for *CA12* from Figure 2

Cancer type	N	Mean	SD	Adjusted *p* value*
Normal Lung	161	4.23	0.55	_
Squamous Cell Carcinoma	46	7.02	1.24	**<0.0001**
Adenocarcinoma	150	5.42	1.48	**<0.0001**
Large Cell Carcinoma	18	4.73	1.28	0.32
Other	48	6.81	1.75	**<0.0001**

*Adjusted *p* values were calculated by Dunnett's multiple comparisons for normal lung (control) versus lung cancer histologies.

Supplementary Table 14: Summary of mRNA data for *CXorf61* from Figure 2

Cancer type	*N*	Mean	SD	Adjusted *p* value*
Normal Lung	161	3.10	0.27	_
Squamous Cell Carcinoma	46	3.54	1.30	0.29
Adenocarcinoma	150	4.86	2.14	**<0.0001**
Large Cell Carcinoma	18	3.53	1.15	0.67
Other	48	4.06	1.87	**0.0005**

*Adjusted *p* values were calculated by Dunnett's multiple comparisons for normal lung (control) versus lung cancer histologies.

Supplementary Table 15: Summary of mRNA data for *DSG3* from Figure 2

Cancer type	*N*	Mean	SD	Adjusted *p* value*
Normal Lung	161	2.90	0.28	_
Squamous Cell Carcinoma	46	8.50	2.19	**<0.0001**
Adenocarcinoma	150	3.31	1.25	0.08
Large Cell Carcinoma	18	3.14	0.90	0.95
Other	48	7.19	3.29	**<0.0001**

*Adjusted *p* values were calculated by Dunnett's multiple comparisons for normal lung (control) versus lung cancer histologies.

Supplementary Table 16: Summary of mRNA data for *FAT2* from Figure 2.

**Cancer type**	***N***	**Mean**	**SD**	**Adjusted *p* value***
Normal Lung	161	3.80	0.24	–
Squamous Cell Carcinoma	46	6.44	1.34	**<0.0001**
Adenocarcinoma	150	3.94	0.68	0.48
Large Cell Carcinoma	18	3.81	0.55	>0.99
Other	48	5.67	1.61	**<0.0001**

*Adjusted *p* values were calculated by Dunnett's multiple comparisons for normal lung (control) versus lung cancer histologies.

Supplementary Table 17: Summary of mRNA data for *GPR87* from Figure 2

**Cancer type**	***N***	**Mean**	**SD**	**Adjusted *p* value***
Normal Lung	161	3.16	0.41	–
Squamous Cell Carcinoma	46	8.01	1.64	**<0.0001**
Adenocarcinoma	150	5.12	2.11	**<0.0001**
Large Cell Carcinoma	18	3.40	1.21	0.95
Other	48	6.77	2.32	**<0.0001**

*Adjusted *p* values were calculated by Dunnett's multiple comparisons for normal lung (control) versus lung cancer histologies.

Supplementary Table 18: Summary of mRNA data for *KISS1R* from Figure 2

**Cancer type**	***N***	**Mean**	**SD**	**Adjusted *p* value***
Normal Lung	161	3.54	0.33	–
Squamous Cell Carcinoma	46	5.26	1.01	**<0.0001**
Adenocarcinoma	150	5.49	1.56	**<0.0001**
Large Cell Carcinoma	18	5.31	2.04	**<0.0001**
Other	48	5.54	1.40	**<0.0001**

*Adjusted *p* values were calculated by Dunnett's multiple comparisons for normal lung (control) versus lung cancer histologies.

Supplementary Table 19: Summary of mRNA data for *LYPD3* from Figure 2

**Cancer type**	***N***	**Mean**	**SD**	**Adjusted *p* value***
Normal Lung	161	5.12	0.33	–
Squamous Cell Carcinoma	46	7.70	1.67	**<0.0001**
Adenocarcinoma	150	5.87	0.97	**<0.0001**
Large Cell Carcinoma	18	5.44	0.55	0.55
Other	48	7.03	1.59	**<0.0001**

*Adjusted *p* values were calculated by Dunnett's multiple comparisons for normal lung (control) versus lung cancer histologies.

Supplementary Table 20: Summary of mRNA data for *SLC7A11* from Figure 2

**Cancer type**	***N***	**Mean**	**SD**	**Adjusted *p* value***
Normal Lung	161	4.14	0.79	–
Squamous Cell Carcinoma	46	7.44	2.00	**<0.0001**
Adenocarcinoma	150	6.12	2.02	**<0.0001**
Large Cell Carcinoma	18	6.66	2.31	**<0.0001**
Other	48	6.78	1.99	**<0.0001**

*Adjusted *p* values were calculated by Dunnett's multiple comparisons for normal lung (control) versus lung cancer histologies.

Supplementary Table 21: Summary of mRNA data for *TMPRSS4* from Figure 2

Cancer type	*N*	Mean	SD	Adjusted *p* value*
Normal Lung	161	4.68	0.51	_
Squamous Cell Carcinoma	46	8.39	1.09	**<0.0001**
Adenocarcinoma	150	6.85	1.32	**<0.0001**
Large Cell Carcinoma	18	5.02	0.95	0.57
Other	48	8.51	1.48	**<0.0001**

*Adjusted *p* values were calculated by Dunnett's multiple comparisons for normal lung (control) versus lung cancer histologies.

Supplementary Table 22: Multiple comparisons between different types of cancer for *CA9* mRNA data from Figure 2*

Cancer type	Adenocarcinoma	Large Cell Carcinoma	Other	Squamous Cell Carcinoma
Adenocarcinoma	_	0.65	**<0.0001**	**0.001**
Large Cell Carcinoma	0.65	_	0.34	0.58
Other	**<0.0001**	0.34	_	0.95
Squamous Cell Carcinoma	**0.001**	0.58	0.95	_

*Adjusted *p* values were calculated by Tukey's all pairwise comparisons.

Supplementary Table 23: Multiple comparisons between different types of cancer for *CA12* mRNA data from Figure 2*

Cancer type	Adenocarcinoma	Large Cell Carcinoma	Other	Squamous Cell Carcinoma
Adenocarcinoma	_	0.25	**<0.0001**	**<0.0001**
Large Cell Carcinoma	0.25	_	**<0.0001**	**<0.0001**
Other	**<0.0001**	**<0.0001**	_	0.90
Squamous Cell Carcinoma	**<0.0001**	**<0.0001**	0.90	_

*Adjusted *p* values were calculated by Tukey's all pairwise comparisons.

Supplementary Table 24: Multiple comparisons between different types of cancer for *CXorf61* mRNA data from Figure 2*

Cancer type	Adenocarcinoma	Large Cell Carcinoma	Other	Squamous Cell Carcinoma
Adenocarcinoma	_	**0.0289**	0.06	**0.0003**
Large Cell Carcinoma	**0.0289**	_	0.75	>0.99
Other	0.06	0.75	_	0.54
Squamous Cell Carcinoma	**0.0003**	>0.99	0.54	_

*Adjusted *p* values were calculated by Tukey's all pairwise comparisons.

Supplementary Table 25: Multiple comparisons between different types of cancer for *DSG3* mRNA data from Figure 2*

Cancer type	Adenocarcinoma	Large Cell Carcinoma	Other	Squamous Cell Carcinoma
Adenocarcinoma	_	0.99	**<0.0001**	**<0.0001**
Large Cell Carcinoma	0.99	_	**<0.0001**	**<0.0001**
Other	**<0.0001**	**<0.0001**	_	**0.0065**
Squamous Cell Carcinoma	**<0.0001**	**<0.0001**	**0.0065**	_

*Adjusted *p* values were calculated by Tukey's all pairwise comparisons.

Supplementary Table 26: Multiple comparisons between different types of cancer for *TMPRSS4* mRNA data from Figure 2[*]

Cancer type	Adenocarcinoma	Large Cell Carcinoma	Other	Squamous Cell Carcinoma
Adenocarcinoma	_	0.96	**<0.0001**	**<0.0001**
Large Cell Carcinoma	0.96	_	**<0.0001**	**<0.0001**
Other	**<0.0001**	**<0.0001**	_	**0.0020**
Squamous Cell Carcinoma	**<0.0001**	**<0.0001**	**0.0020**	_

[*]Adjusted *p* values were calculated by Tukey's all pairwise comparisons.

Supplementary Table 27: Multiple comparisons between different types of cancer for *GPR87* mRNA data from Figure 2[*]

Cancer type	Adenocarcinoma	Large Cell Carcinoma	Other	Squamous Cell Carcinoma
Adenocarcinoma	–	**0.0044**	**<0.0001**	**<0.0001**
Large Cell Carcinoma	**0.0044**	–	**<0.0001**	**<0.0001**
Other	**<0.0001**	**<0.0001**	–	**0.0172**
Squamous Cell Carcinoma	**<0.0001**	**<0.0001**	**0.0172**	–

[*]Adjusted *p* values were calculated by Tukey's all pairwise comparisons.

Supplementary Table 28: Multiple comparisons between different types of cancer for *KISS1R* mRNA data from Figure 2[*]

Cancer type	Adenocarcinoma	Large Cell Carcinoma	Other	Squamous Cell Carcinoma
Adenocarcinoma	–	0.96	>0.99	0.80
Large Cell Carcinoma	0.96	–	0.94	>0.99
Other	>0.99	0.94	–	0.80
Squamous Cell Carcinoma	0.80	>0.99	0.80	–

[*]Adjusted *p* values were calculated by Tukey's all pairwise comparisons.

Supplementary Table 29: Multiple comparisons between different types of cancer for *LYPD3* mRNA data from Figure 2[*]

Cancer type	Adenocarcinoma	Large Cell Carcinoma	Other	Squamous Cell Carcinoma
Adenocarcinoma	–	0.50	**<0.0001**	**<0.0001**
Large Cell Carcinoma	0.50	–	**<0.0001**	**<0.0001**
Other	**<0.0001**	**<0.0001**	–	**0.0434**
Squamous Cell Carcinoma	**<0.0001**	**<0.0001**	**0.0434**	–

[*]Adjusted *p* values were calculated by Tukey's all pairwise comparisons.

Supplementary Table 30: Multiple comparisons between different types of cancer for *SLC7A11* mRNA data from Figure 2[*]

Cancer type	Adenocarcinoma	Large Cell Carcinoma	Other	Squamous Cell Carcinoma
Adenocarcinoma	–	0.70	0.20	**0.0008**
Large Cell Carcinoma	0.70	–	>0.99	0.52
Other	0.20	>0.99	–	0.40
Squamous Cell Carcinoma	**0.0008**	0.52	0.40	–

[*]Adjusted *p* values were calculated by Tukey's all pairwise comparisons.

Supplementary Table 31: Multiple comparisons between different types of cancer for *TMPRSS4* mRNA data from Figure 2*

Cancer type	Adenocarcinoma	Large Cell Carcinoma	Other	Squamous Cell Carcinoma
Adenocarcinoma	–	**<0.0001**	**<0.0001**	**<0.0001**
Large Cell Carcinoma	**<0.0001**	–	**<0.0001**	**<0.0001**
Other	**<0.0001**	**<0.0001**	–	0.97
Squamous Cell Carcinoma	**<0.0001**	**<0.0001**	0.97	–

*Adjusted *p* values were calculated by Tukey's all pairwise comparisons.

Supplementary Table 32: Immunohistochemical scoring of marker expression in other normal patient tissue samples

Target*	Tissue type	Patient Tissue Samples (*n*)	Pathology Score				Heterogeneity Score# (Average ± SD)
			0	1+	2+	3+	
**CA9**	**Liver**	**3**	100%	0%	0%	0%	100% ± 0%
	**Spleen**	**5**	0%	40%	0%	60%	60% ± 30%
	**Lymph Node**	**2**	0%	0%	50%	50%	70% ± 28%
**CA12**	**Liver**	**4**	50%	50%	0%	0%	100% ± 0%
	**Spleen**	**6**	0%	67%	33%	0%	87% ± 21%
	**Lymph Node**	**2**	100%	0%	0%	0%	100% ± 0%
**CXorf61**	**Liver**	**4**	100%	0%	0%	0%	100% ± 0%
	**Spleen**	**6**	0%	67%	33%	0%	100% ± 0%
	**Lymph Node**	**2**	0%	50%	50%	0%	100% ± 0%
**DSG3**	**Liver**	**4**	50%	50%	0%	0%	100% ± 0%
	**Spleen**	**6**	0%	33%	67%	0%	100% ± 0%
	**Lymph Node**	**2**	0%	0%	0%	100%	100% ± 0%
**FAT2**	**Liver**	**4**	0%	0%	50%	50%	100% ± 0%
	**Spleen**	**6**	0%	100%	0%	0%	88% ± 10%
	**Lymph Node**	**2**	0%	100%	0%	0%	100% ± 0%
**GPR87**	**Liver**	**4**	50%	50%	0%	0%	15% ± 7%
	**Spleen**	**6**	0%	0%	100%	0%	77% ± 36%
	**Lymph Node**	**2**	0%	0%	50%	50%	70% ± 0%
**KISS1R**	**Liver**	**4**	0%	0%	50%	50%	100% ± 0%
	**Spleen**	**6**	0%	0%	100%	0%	100% ± 0%
	**Lymph Node**	**2**	0%	0%	100%	0%	100% ± 0%
**LYPD3**	**Liver**	**4**	50%	50%	0%	0%	50% ± 0%
	**Spleen**	**6**	0%	67%	33%	0%	90% ± 15%
	**Lymph Node**	**2**	0%	100%	0%	0%	70% ± 0%
**SLC7A11**	**Liver**	**4**	100%	0%	0%	0%	100% ± 0%
	**Spleen**	**6**	100%	0%	0%	0%	100% ± 0%
	**Lymph Node**	**2**	100%	0%	0%	0%	100% ± 0%
**TMPRSS4**	**Liver**	**4**	100%	0%	0%	0%	100% ± 0%
	**Spleen**	**6**	0%	83%	17%	0%	100% ± 0%
	**Lymph Node**	**2**	50%	0%	50%	0%	100%

*Protein expression is scored but gene names are used to conserve space.

#Heterogeneity score indicates the average percentage of cell staining in samples that stained regardless of pathology score. For samples with pathology scores of 0 only, 100% heterogeneity score indicates uniformly unstained.

Supplementary Table 33: List of datasets used in tissue analyses. See Supplementary_Table_33

Supplementary Table 34: Sample annotation and metadata for all tissue samples used in analyses. See Supplementary_Table_34

Supplementary Table 35: List of potential surface accessible probesets. See Supplementary_Table_35

Supplementary Table 36: Demographics of the patient cohort whose samples were included in the lung cancer tissue microarray (TMA) used for immunohistochemistry (IHC)

**Age at Diagnosis**	***N***	**%**
30–39	2	1.98
40–49	6	5.94
50–59	30	29.70
60–69	42	41.58
70–79	21	20.79
**Sex**	***N***	**%**
FEMALE	46	45.54
MALE	55	54.46
**Race**	***N***	**%**
OTHER	1	0.99
WHITE	100	99.01
**Ethnicity**	***N***	**%**
NON-SPANISH/NON-HISPANIC	97	96.04
SPANISH/HISPANIC	4	3.96
**Histology**	***N***	**%**
Adenocarcinoma	66	65.35
Squamous Cell Carcinoma	10	9.90
Large Cell Carcinoma	3	2.97
NSCLC/Other	22	21.78
**Pathological TNM Stage**	***N***	**%***
I	5	5.38
IA	8	8.60
IB	19	20.43
IIA	1	1.08
IIB	9	9.68
III	1	1.08
IIIA	16	17.20
IIIB	10	10.75
IV	24	25.81
Missing/Unknown	8	
**Smoking Status**	***N***	**%***
EVER	92	92.93%
NEVER	7	7.07%
MISSING	2	

*Percentages are calculated using non-missing values only.

Supplementary Table 37: Antibodies, control tissues, and conditions used for immunohistochemistry (IHC)

Antibody*	Product	Species	Control Tissue (IHC)	Antibody Dilution	Antibody Incubation Time (Primary, Secondary[a])	Antigen Retrieval
CA9	Ab15086, Abcam	Polyclonal Rabbit	Kidney tumor	1:500	32 min, 20 min	RiboCC
CA12	HPA008773, Sigma Aldrich	Polyclonal Rabbit	Normal kidney	1:75	32 min, 20 min	Cell Conditioning 1
CXorf61	HPA004773, Sigma Aldrich	Polyclonal Rabbit	Colon cancer and Brain	1:25	8 hours, 12 min	RiboCC
DSG3	NBP1-78984, Novus Biologicals	Monoclonal Mouse	Skin	1:200	3 hours, 16 min	RiboCC
FAT2	sc-59985, Santa Cruz	Monoclonal Mouse	Bladder Cancer	1:25	60 min, 16 min	Cell Conditioning 1
GPR87	IMG-71566, Imgenex	Polyclonal Rabbit	Prostate	1:75	32 min, 16 min	RiboCC
LYPD3	NBP1-44371, Novus Biologicals	Polyclonal Rabbit	Skin	1:50	32 min, 16 min	RiboCC
KISS1R (GPR54)	IMG-71415, Imgenex	Polyclonal Rabbit	Normal Pancreas	1:50	3 hours, 8 min	Cell Conditioning 1
SLC7A11 (xCT)	NB300-318, Novus Biologicals	Polyclonal Rabbit	Small Bowel	1:500	60 min, 16 min	Cell Conditioning 1
TMPRSS4	11283-1-AP, ProteinTech	Polyclonal Rabbit	Pancreatic Cancer	1:75	60 min, 16 min	RiboCC

*Gene names are used for brevity. [a]The appropriate anti-rabbit or anti-mouse secondary antibody was used. The Ventana Omnimap secondary antibodies were used for all of the markers except for CXorf61 and GPR87. The Ventana Ultramap secondary antibody was used for CXorf61 and GPR87.

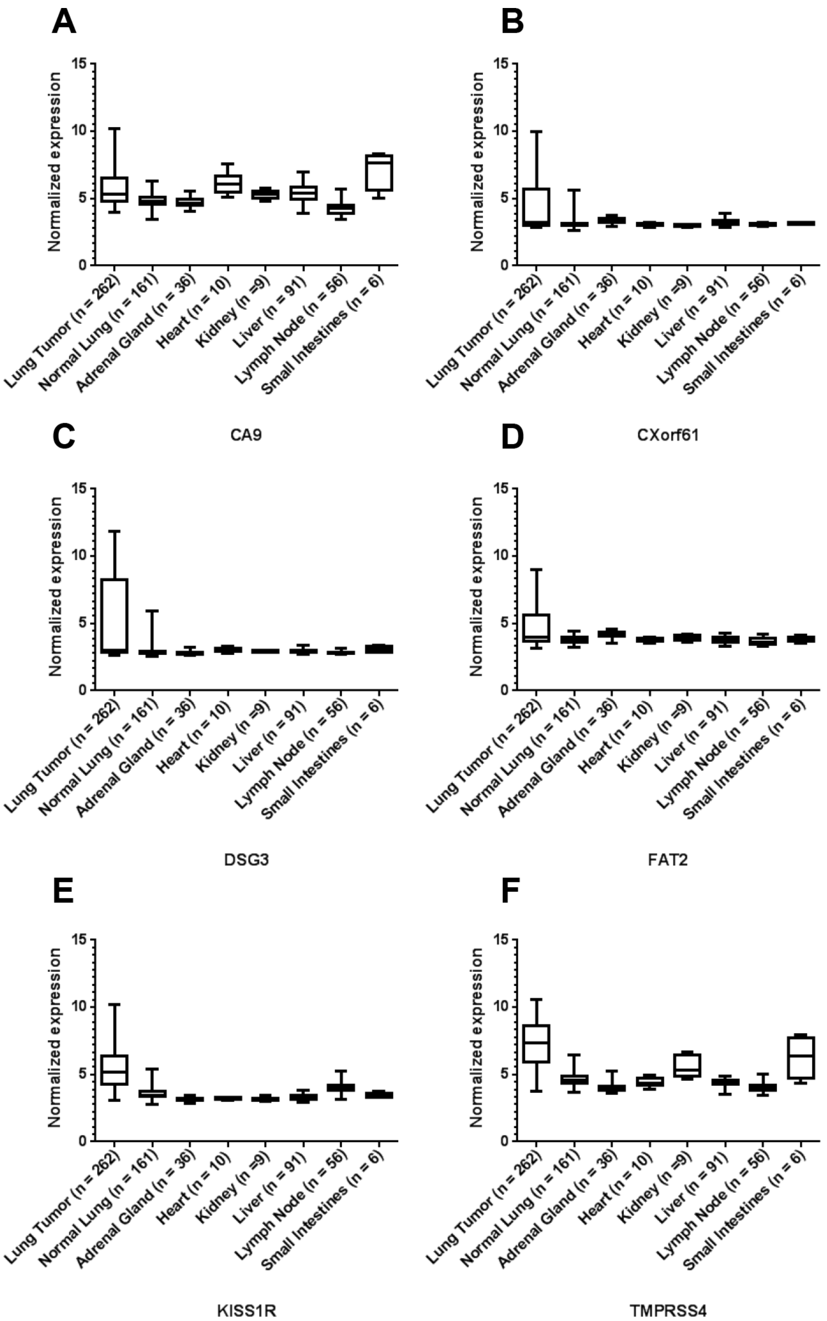

Supplementary Figure 1: Representative microarray mRNA expression profiles for six of the selected lung cancer cell-surface markers in patient specimens of normal lung, lung tumors and other normal tissues: CA9 (**A**), CXorf61 (**B**), DSG3 (**C**), FAT2 (**D**), KISS1R (**E**), and TMPRSS4 (**F**). Values are presented as whisker/box plots with whiskers representing the full range of values, the bottom and top of the boxes represent the 25th and 75th percentile, and middle lines represent the median.

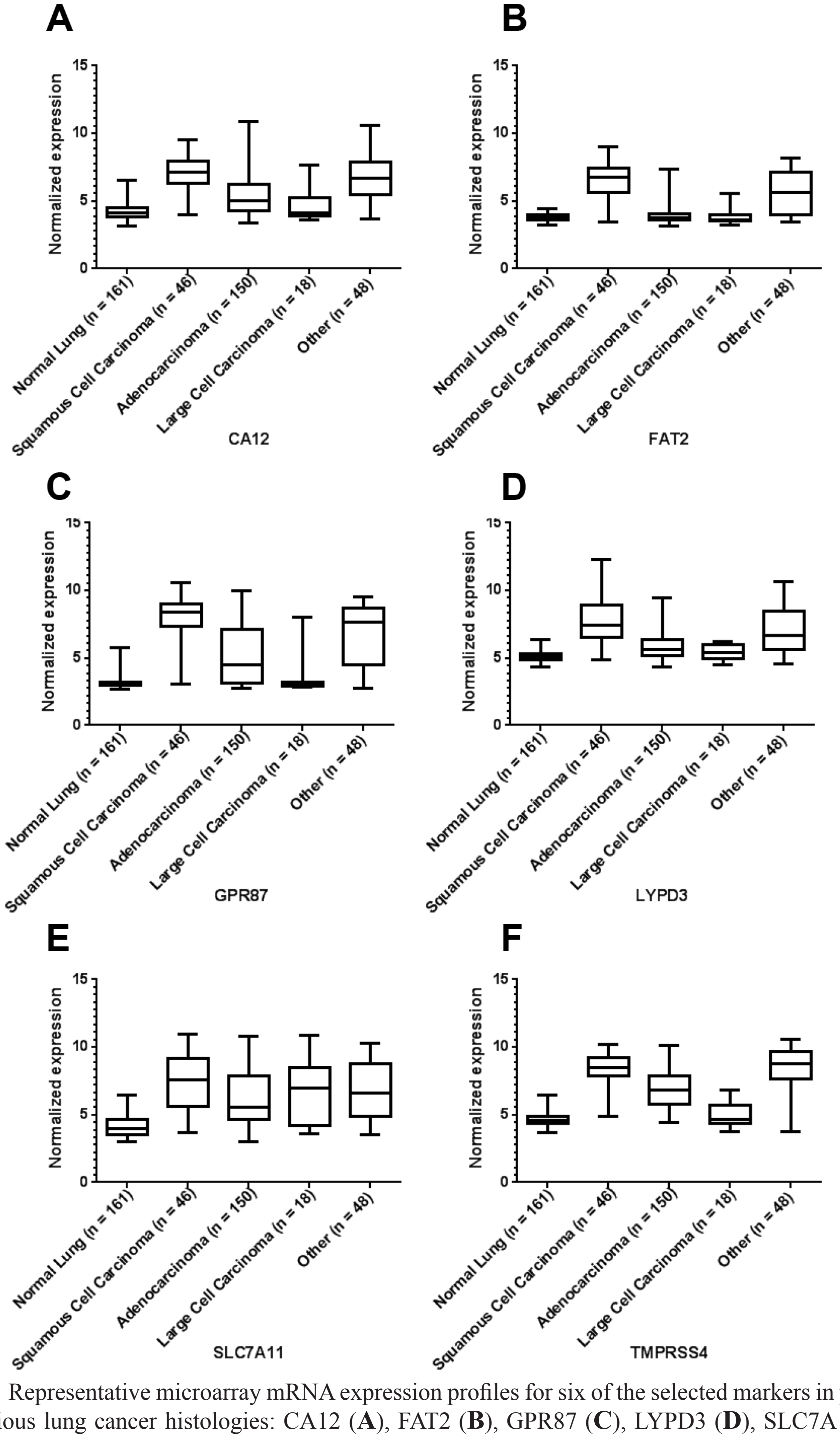

Supplementary Figure 2: Representative microarray mRNA expression profiles for six of the selected markers in patient specimens of normal lung and lung tumors of various lung cancer histologies: CA12 (**A**), FAT2 (**B**), GPR87 (**C**), LYPD3 (**D**), SLC7A11 (**E**), and TMPRSS4 (**F**). Values are presented as whisker/box plots with whiskers representing the full range of values, the bottom and top of the boxes represent the 25th and 75th percentile, and the middle lines represent the median.

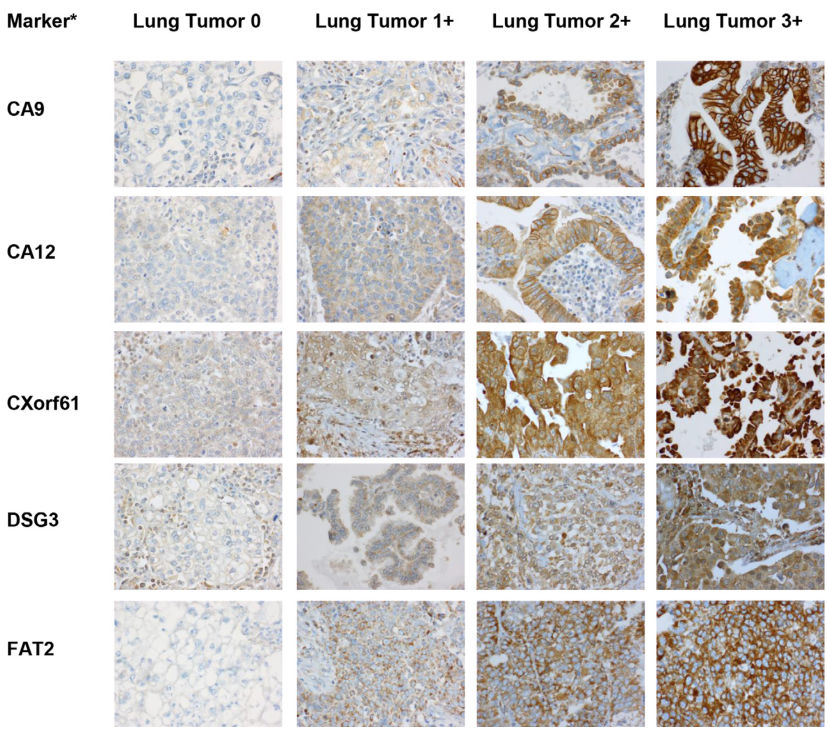

Supplementary Figure 3: Representative images of IHC stained patient lung tumor specimens from the tissue microarray (TMA) for half of the selected markers. Representative lung tumor samples with scores of 0, 1+, 2+, and 3+ are shown for each marker. The images are taken at 40x magnification. *Protein expression is stained but gene names are used to conserve space.

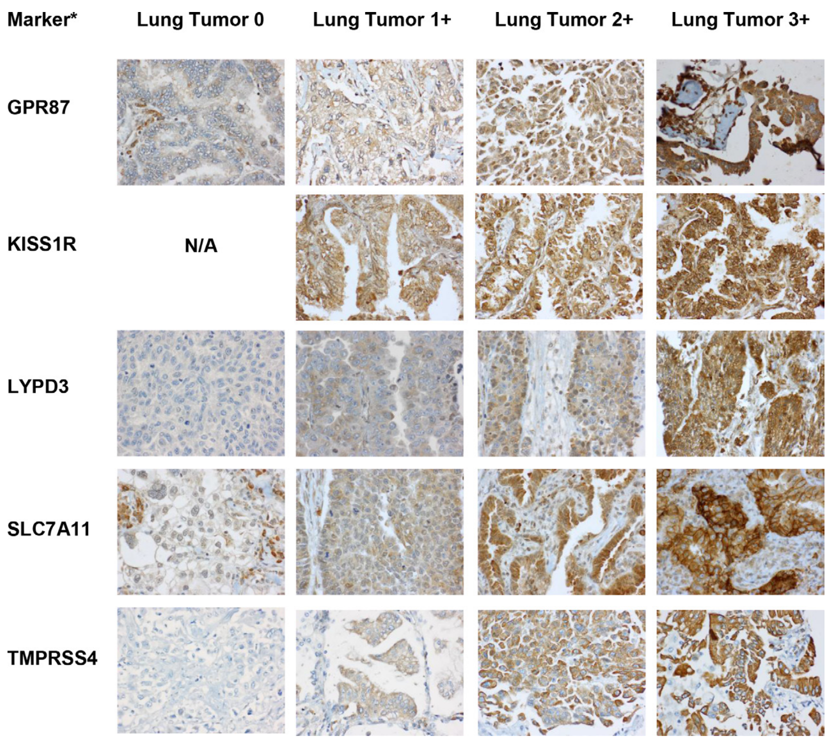

Supplementary Figure 4: Representative images of IHC stained patient lung tumor specimens from the tissue microarray (TMA) for the remaining selected markers. Representative lung tumor samples with scores of 0, 1+, 2+, and 3+ are shown for each marker. The images are taken at 40x magnification. *Protein expression is stained but gene names are used to conserve space.

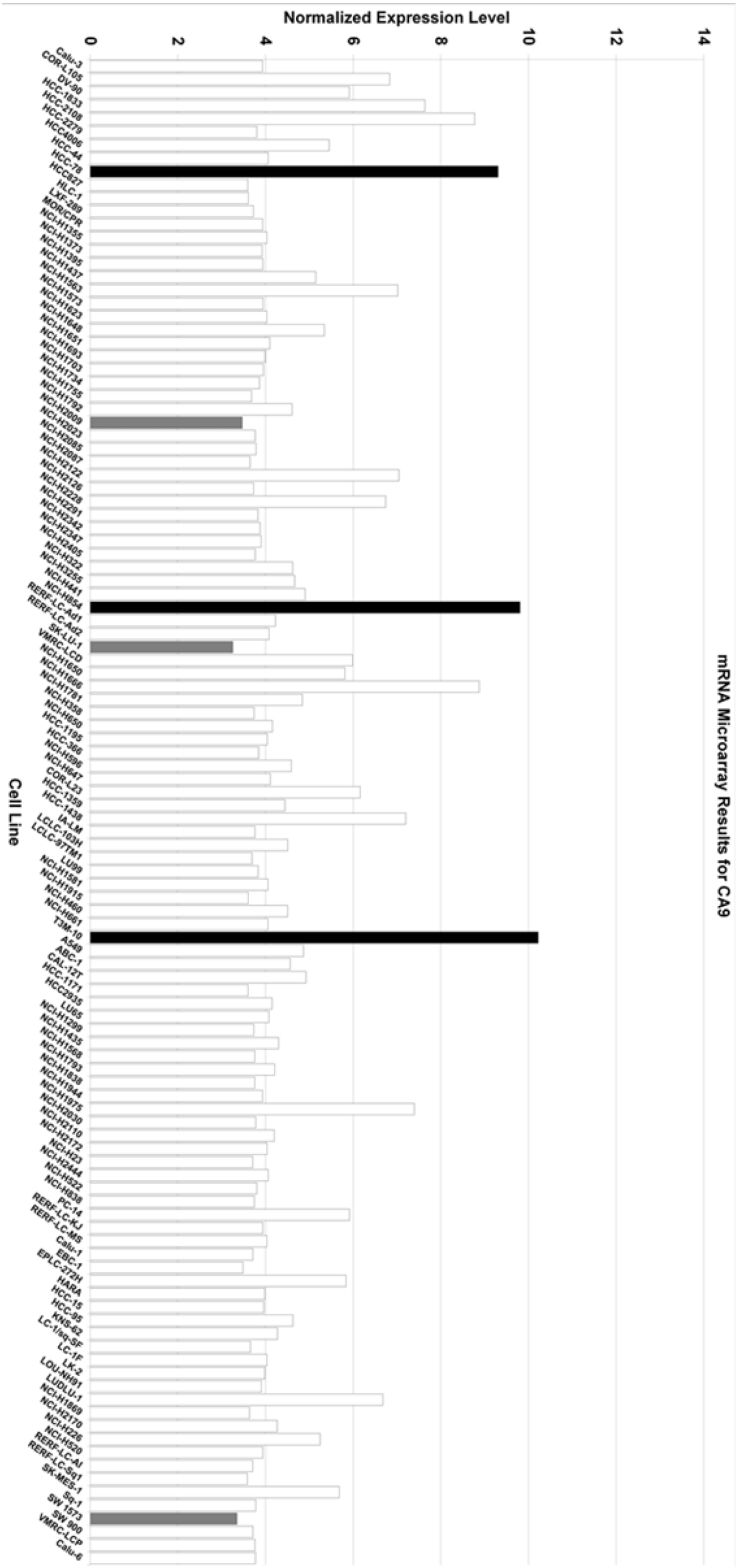

Supplementary Figure 5: mRNA microarray data for the expression of the *CA9* gene in cell lines. Normalized expression of *CA9* based on Affymetrix microarray data. *CA9* expression was analyzed for a panel of lung cancer cell lines. The data for the high-expressing and low/non-expressing cell lines are shaded black and gray respectively.

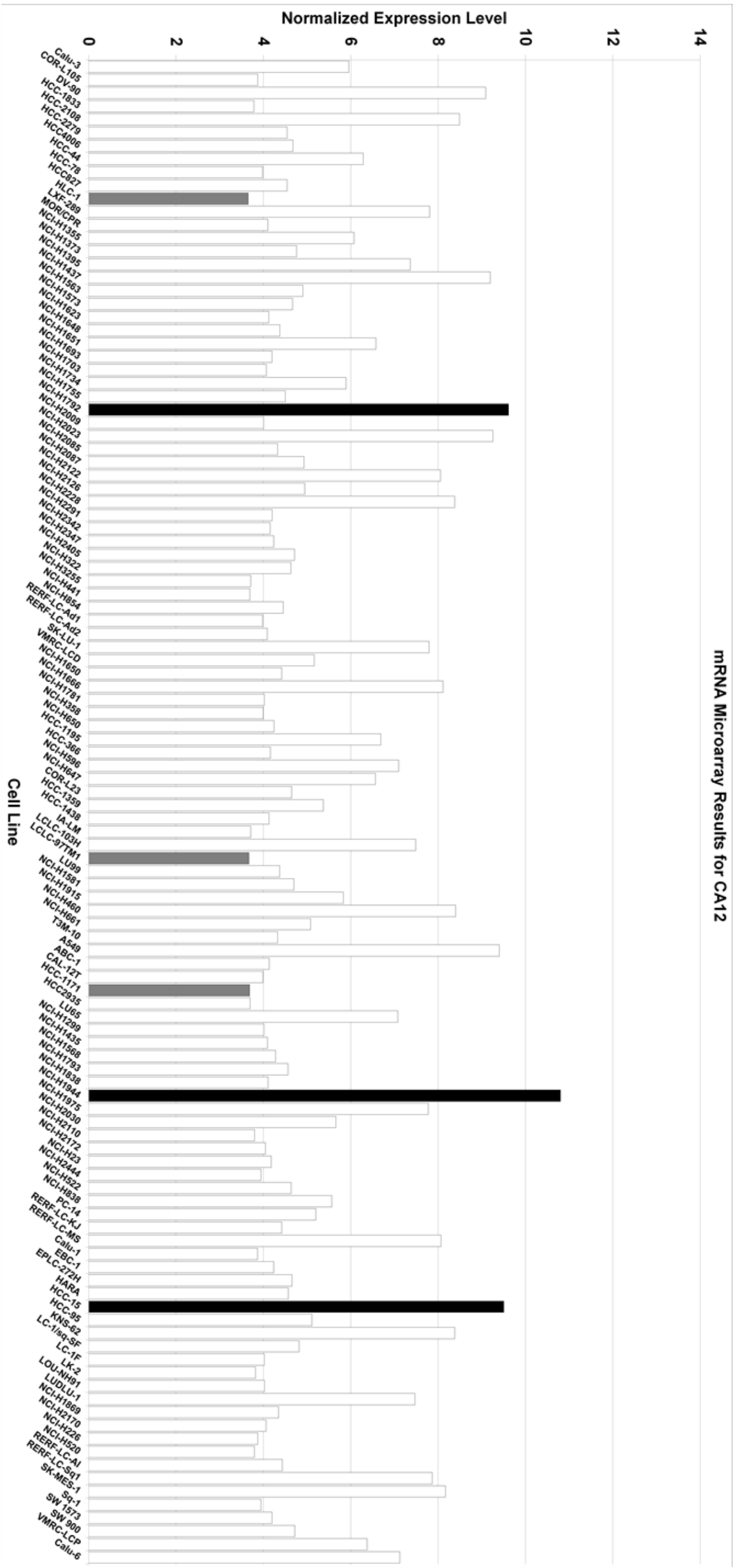

Supplementary Figure 6: mRNA microarray data for the expression of the *CA12* gene in cell lines. Normalized expression of *CA12* based on Affymetrix microarray data. *CA12* expression was analyzed for a panel of lung cancer cell lines. The data for the high-expressing and low/non-expressing cell lines are shaded black and gray respectively.

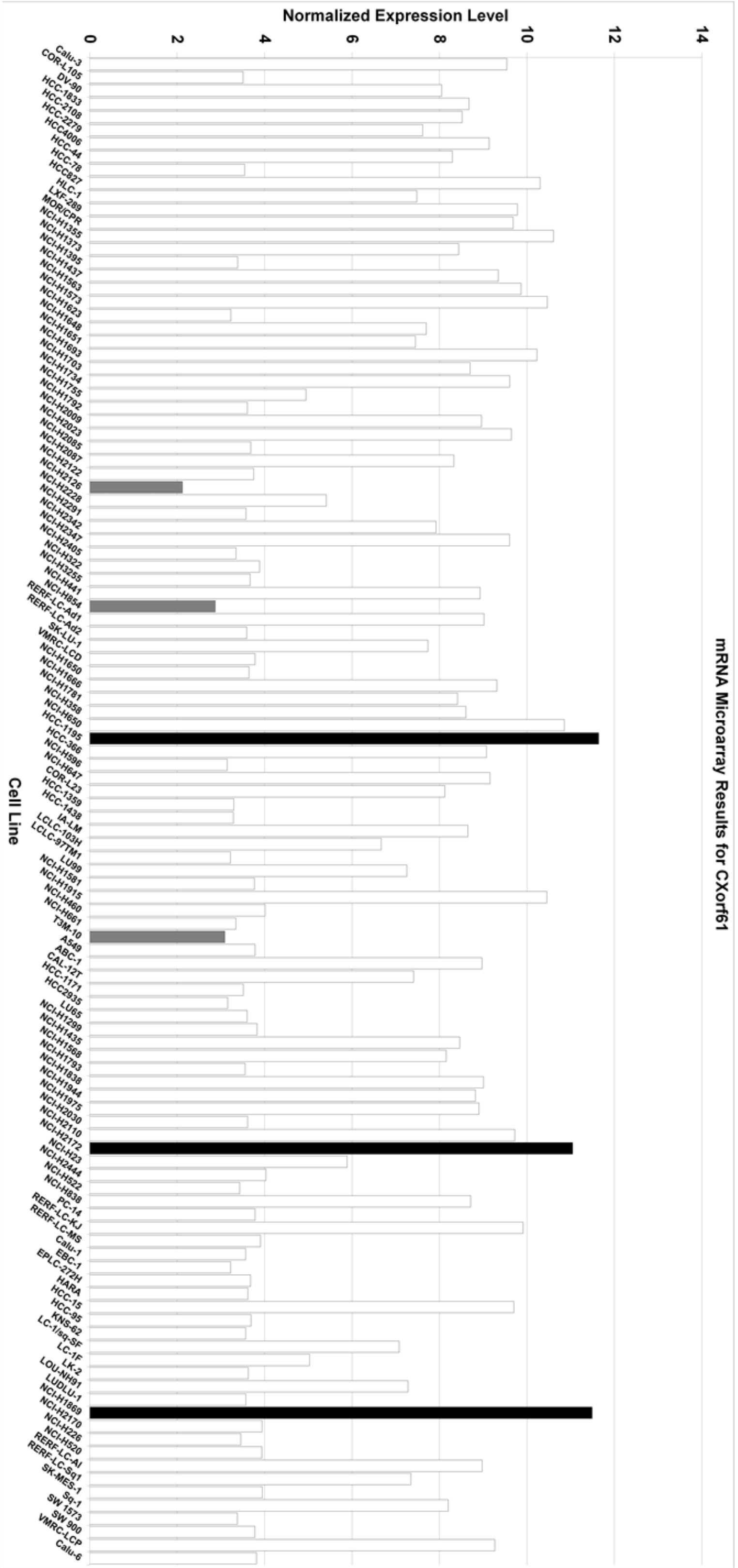

Supplementary Figure 7: mRNA microarray data for the expression of the *CXorf61* gene in cell lines. Normalized expression of *CXorf61* based on Affymetrix microarray data. *CXorf61* expression was analyzed for a panel of lung cancer cell lines. The data for the high-expressing and low/non-expressing cell lines are shaded black and gray respectively.

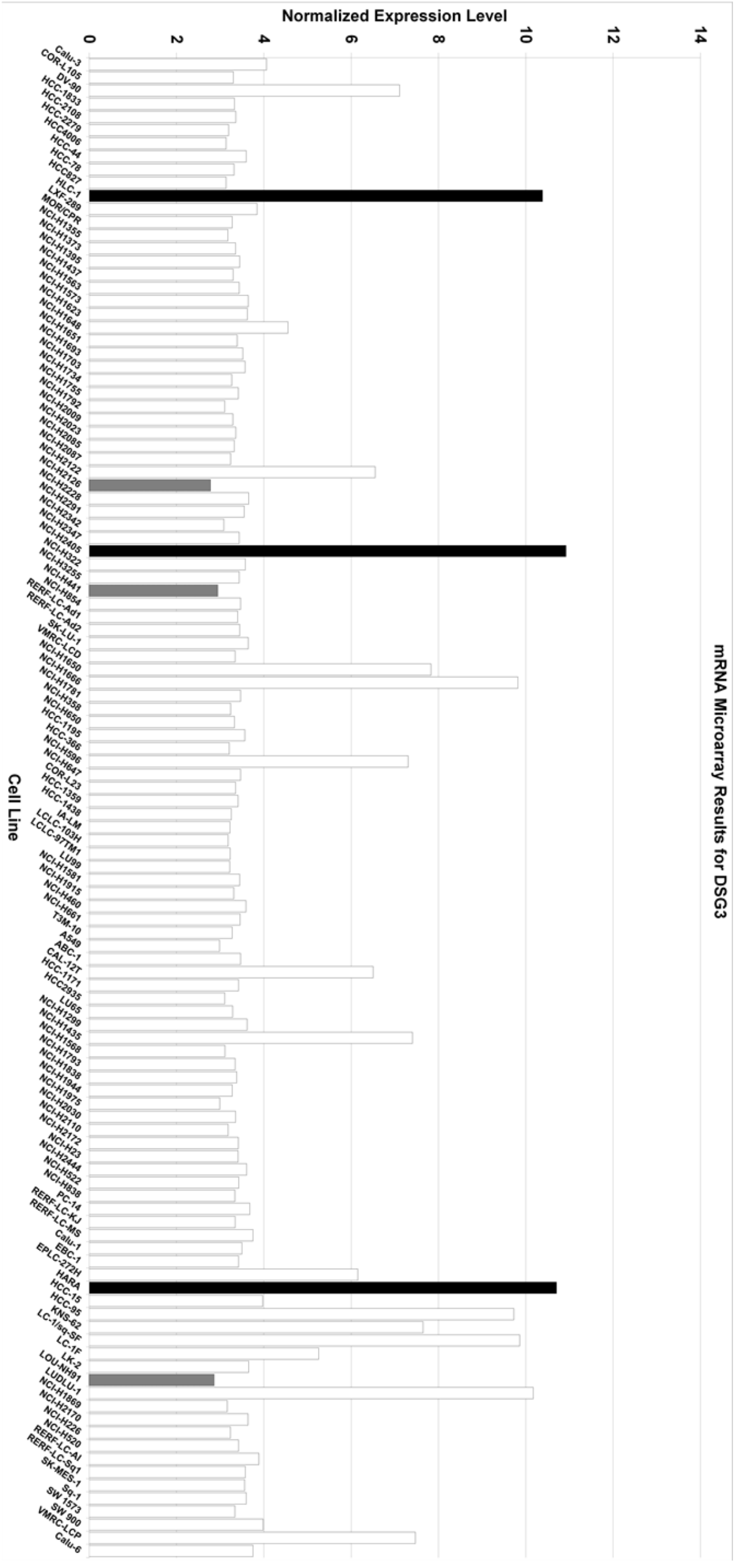

Supplementary Figure 8: mRNA microarray data for the expression of the *DSG3* gene in cell lines. Normalized expression of *DSG3* based on Affymetrix microarray data. *DSG3* expression was analyzed for a panel of lung cancer cell lines. The data for the high-expressing and low/non-expressing cell lines are shaded black and gray respectively.

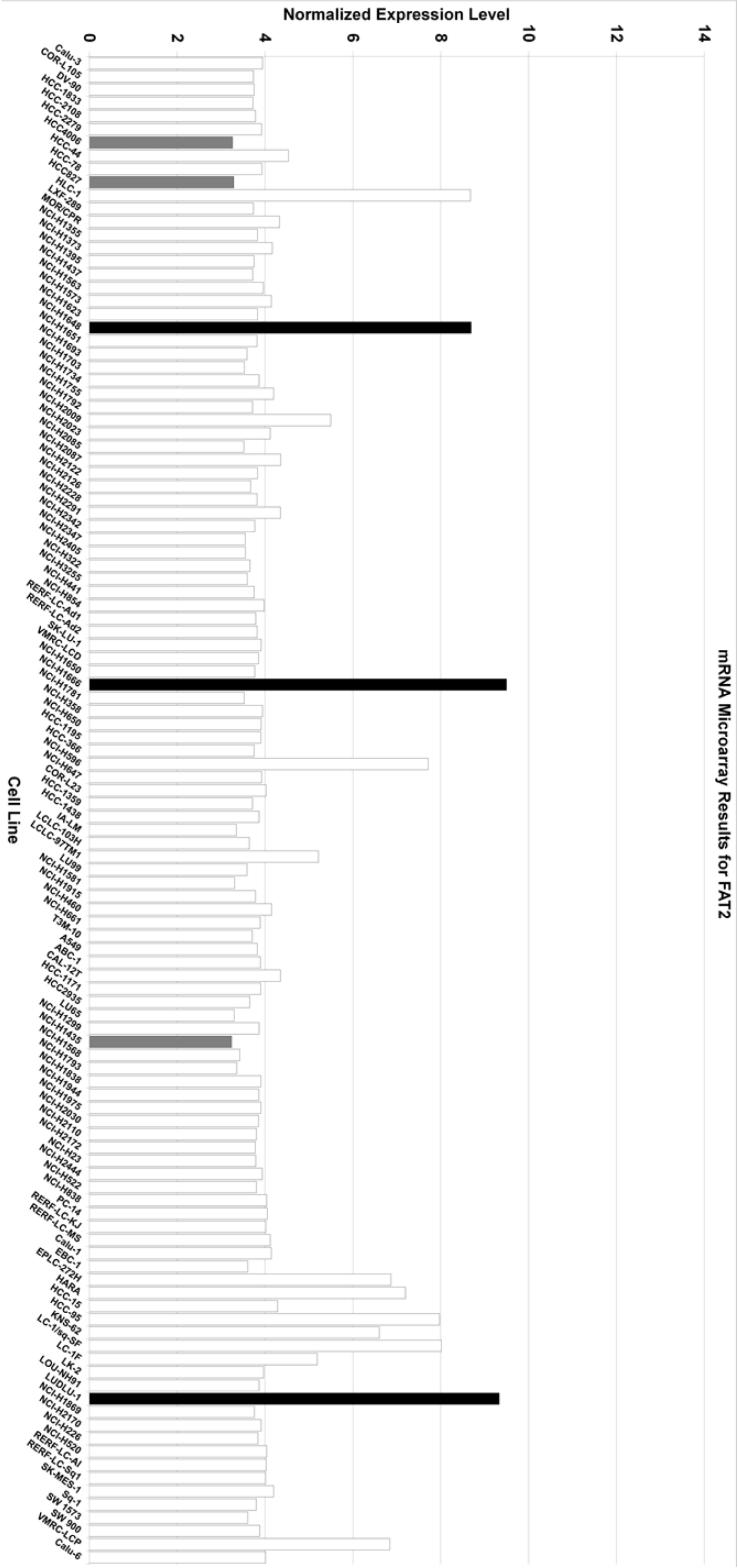

Supplementary Figure 9: mRNA microarray data for the expression of the *FAT2* gene in cell lines. Normalized expression of *FAT2* based on Affymetrix microarray data. *FAT2* expression was analyzed for a panel of lung cancer cell lines. The data for the high-expressing and low/non-expressing cell lines are shaded black and gray respectively.

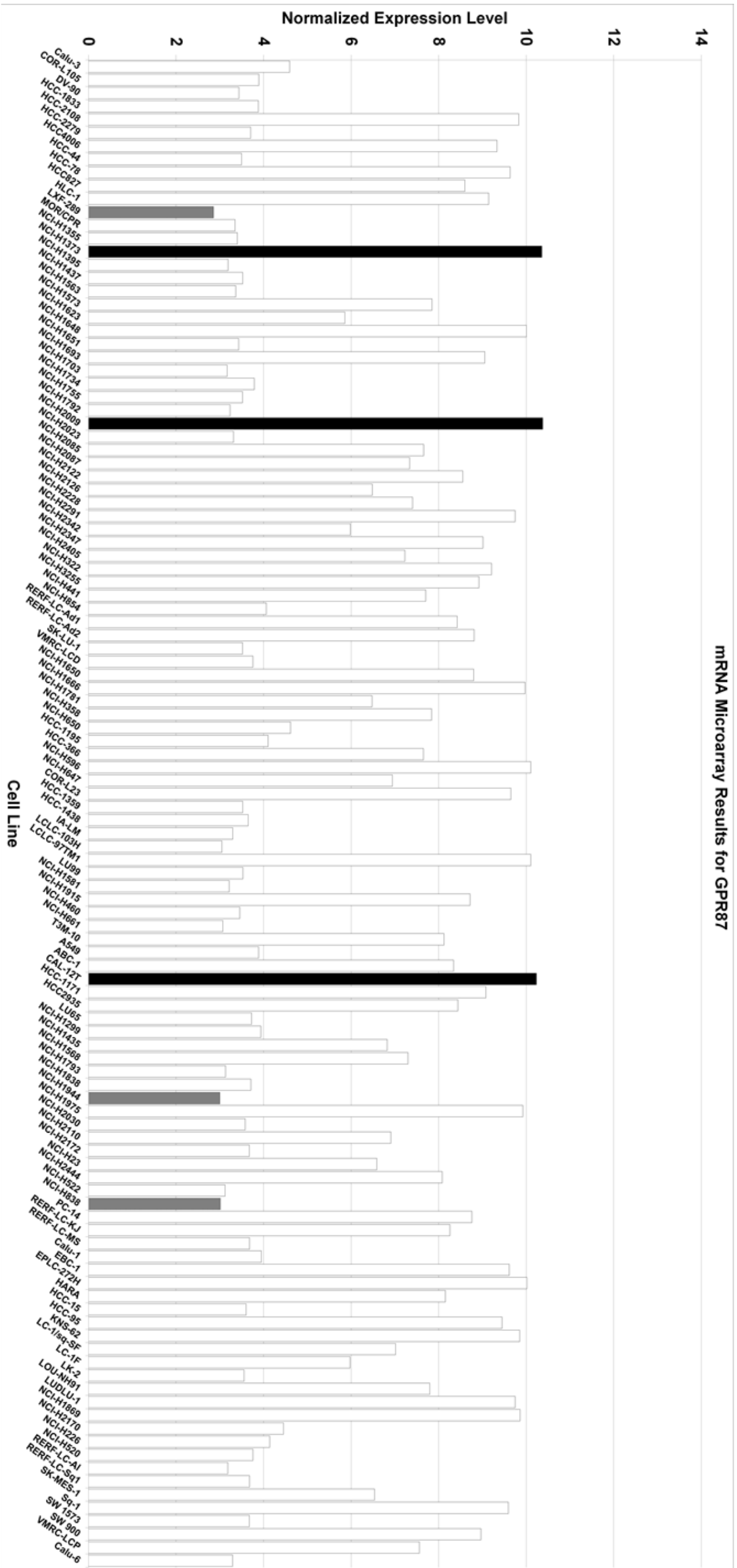

Supplementary Figure 10: mRNA microarray data for the expression of the *GPR87* gene in cell lines. Normalized expression of *GPR87* based on Affymetrix microarray data. *GPR87* expression was analyzed for a panel of lung cancer cell lines. The data for the high-expressing and low/non-expressing cell lines are shaded black and gray respectively.

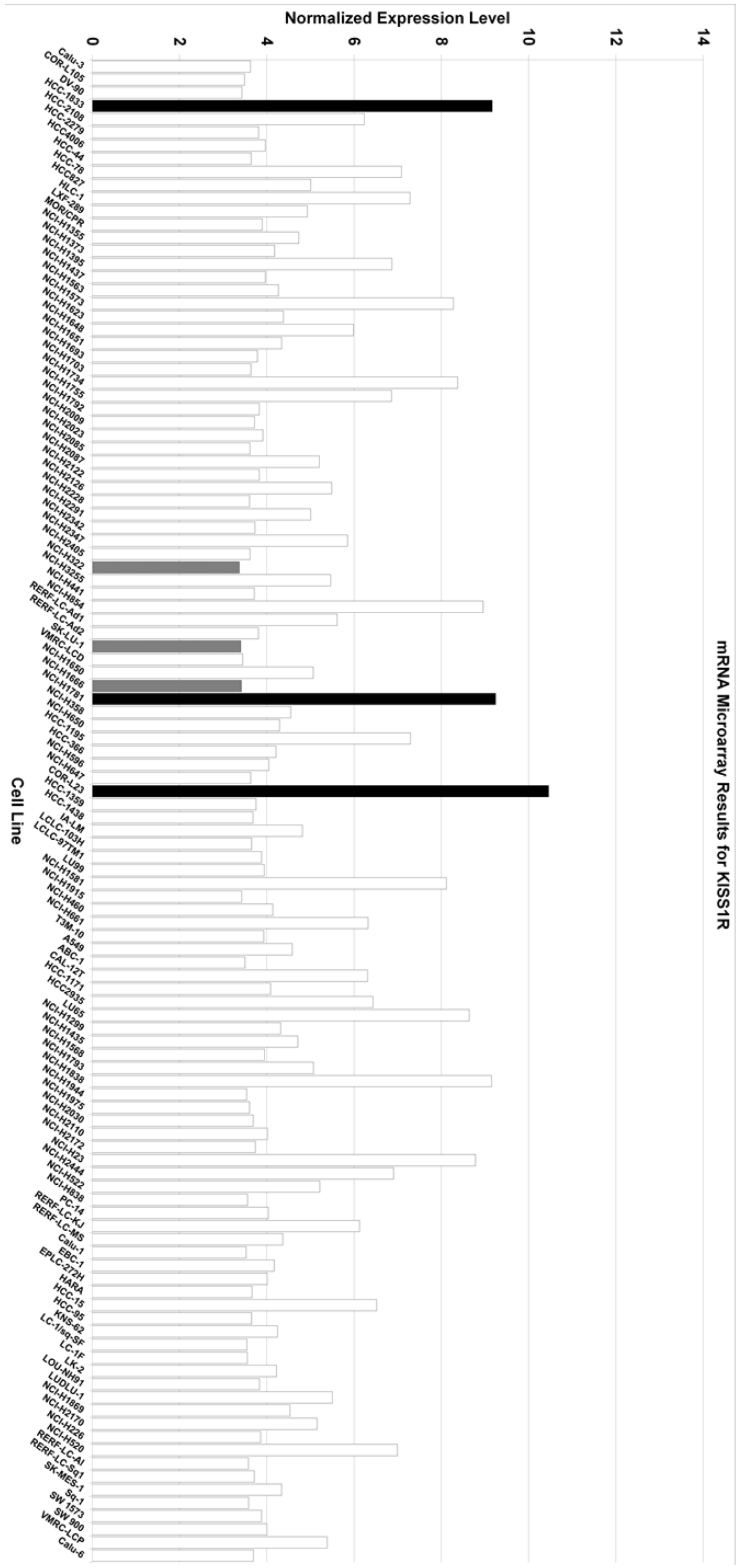

Supplementary Figure 11: mRNA microarray data for the expression of the *KISS1R* gene in cell lines. Normalized expression of *KISS1R* based on Affymetrix microarray data. *KISS1R* expression was analyzed for a panel of lung cancer cell lines. The data for the high-expressing and low/non-expressing cell lines are shaded black and gray respectively.

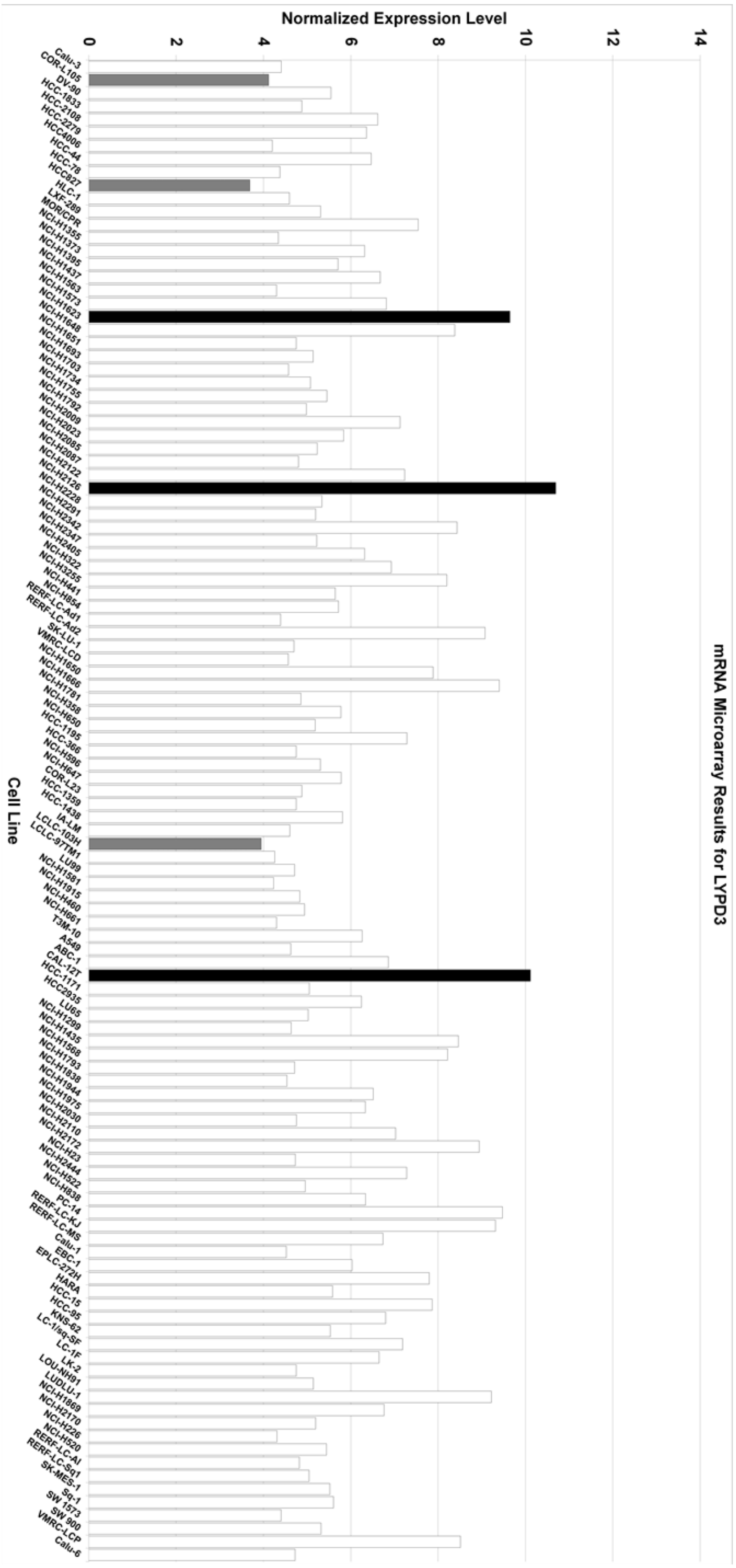

Supplementary Figure 12: mRNA microarray data for the expression of the *LYPD3* gene in cell lines. Normalized expression of *LYPD3* based on Affymetrix microarray data. *LYPD3* expression was analyzed for a panel of lung cancer cell lines. The data for the high-expressing and low/non-expressing cell lines are shaded black and gray respectively.

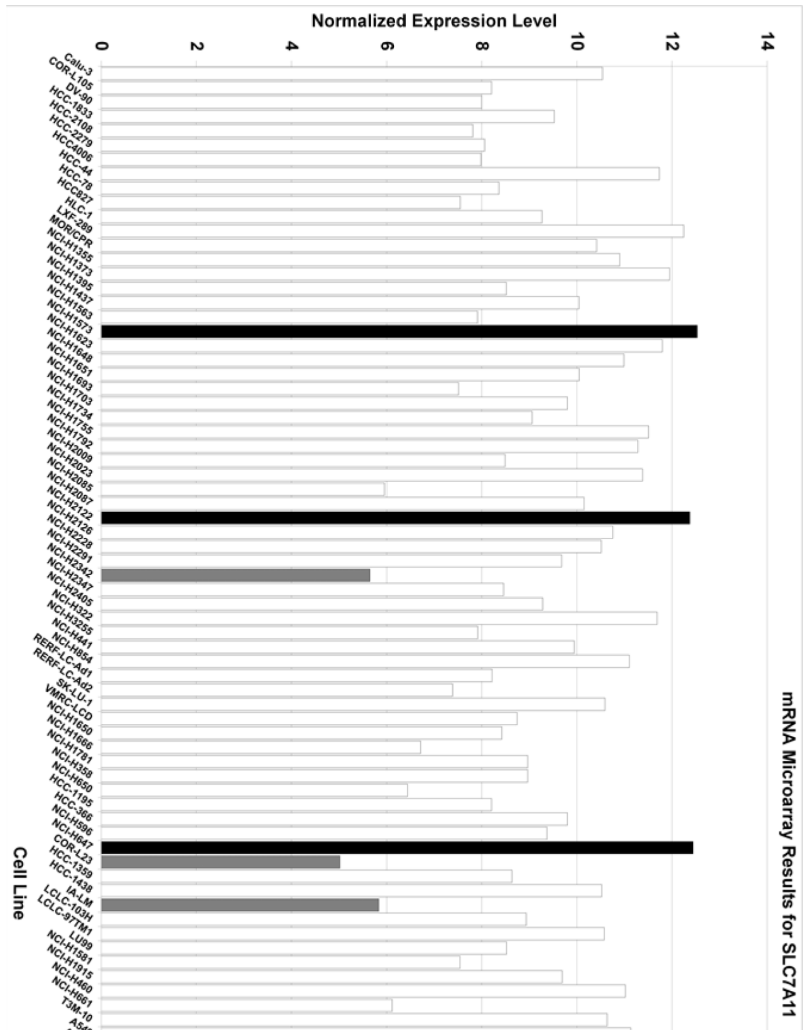

Supplementary Figure 13: mRNA microarray data for the expression of the *SLC7A11* gene in cell lines. Normalized expression of *SLC7A11* based on Affymetrix microarray data. *SLC7A11* expression was analyzed for a panel of lung cancer cell lines. The data for the high-expressing and low/non-expressing cell lines are shaded black and gray respectively.

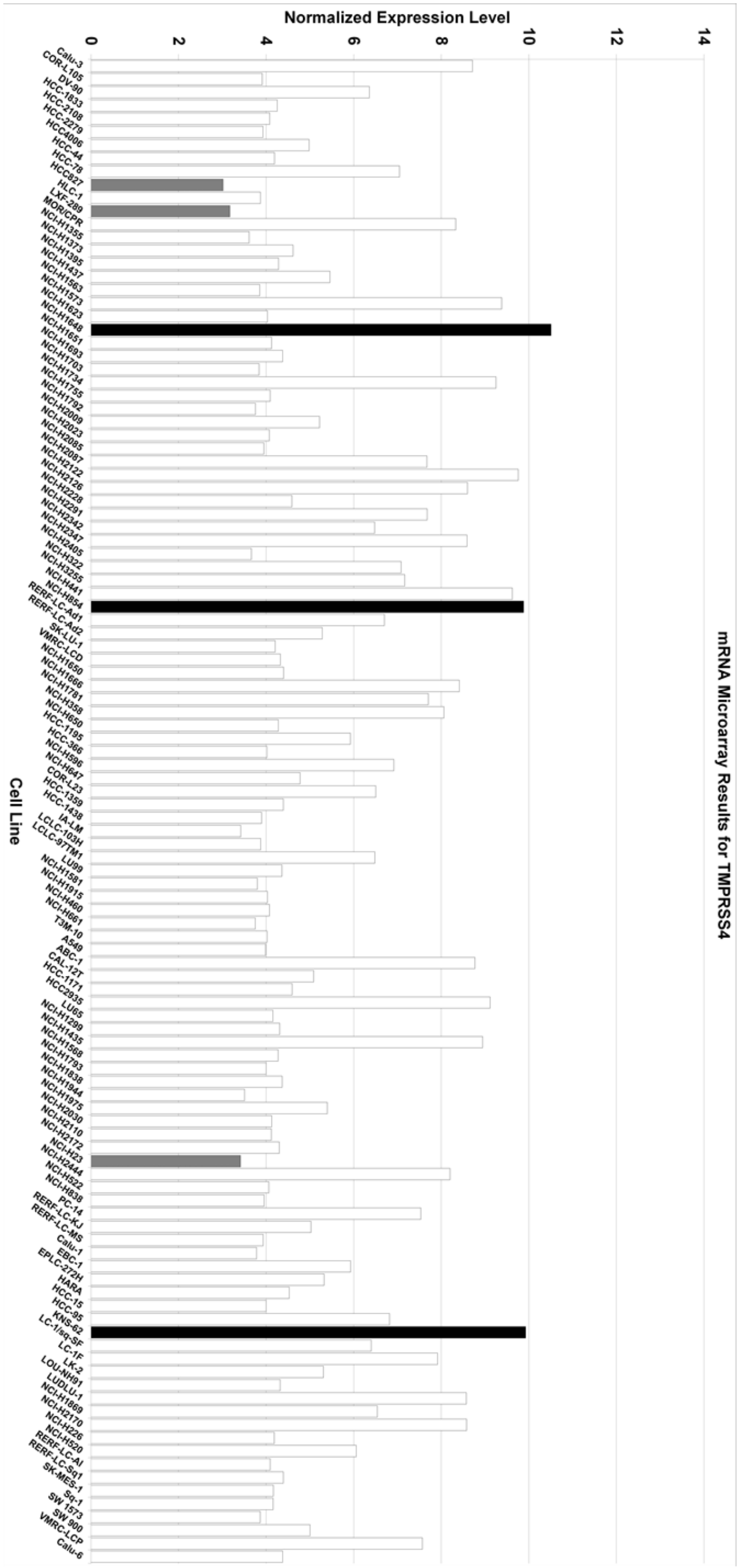

Supplementary Figure 14: mRNA microarray data for the expression of the *TMPRSS4* gene in cell lines. Normalized expression of *TMPRSS4* based on Affymetrix microarray data. *TMPRSS4* expression was analyzed for a panel of lung cancer cell lines. The data for the high-expressing and low/non-expressing cell lines are shaded black and gray respectively.
